# What a Difference an Amino Acid Makes: An All-Atom Simulation Study of Nonameric Peptides in Inhibitory HLA-E/NKG2A/CD94 Immune Complexes

**DOI:** 10.3389/fphar.2022.925427

**Published:** 2022-08-04

**Authors:** Eva Prašnikar, Andrej Perdih, Jure Borišek

**Affiliations:** ^1^ Theory Department, Laboratory for Chemical Informatics, National Institute of Chemistry, Ljubljana, Slovenia; ^2^ Faculty of Medicine, Graduate School of Biomedicine, University of Ljubljana, Ljubljana, Slovenia; ^3^ Theory Department, Laboratory for Computational Biochemistry and Drug Design, National Institute of Chemistry, Ljubljana, Slovenia; ^4^ Faculty of Pharmacy, University of Ljubljana, Ljubljana, Slovenia

**Keywords:** immune system checkpoint, peptide design, HLA-E, NK cell, T-cell

## Abstract

MHC class I antigen E (HLA-E), a ligand for the inhibitory NKG2A/CD94 receptor of the immune system, is responsible for evading the immune surveillance in several settings, including senescent cell accumulation and tumor persistence. The formation of this ligand-receptor interaction promotes the inhibition of the cytolytic action of immune system natural killer (NK) cells and CD8^+^ T-cells expressing this receptor. The final outcome of the HLA-E/NKG2A/CD94 interaction on target cells is also highly dependent on the identity of the nonameric peptide incorporated into the HLA-E ligand. To better understand the role played by a nonameric peptide in these immune complexes, we performed a series of multi-microsecond all-atom molecular dynamics simulations. We generated natural and alternative variants of the nonameric peptide bound to the HLA-E ligand alone or in the HLA-E/NKG2A/CD94 complexes. A systematic study of molecular recognition between HLA-E and peptides led to the development of new variants that differ at the strategic 6th position (P6) of the peptide and have favorable *in silico* properties comparable to those of natural binding peptides. Further examination of a selected subset of peptides in full complexes revealed a new variant that, according to our previously derived atomistic model, can interfere with the signal transduction *via* HLA-E/NKG2A/CD94 and thus prevent the target cell from evading immune clearance by NK and CD8^+^ T-cells. These simulations provide an atomistic picture of how a small change in amino acid sequence can lead to a profound effect on binding and molecular recognition. Furthermore, our study also provides new data on the peptide interaction motifs as well as the energetic and conformational properties of the binding interface, laying the structure-based foundation for future development of potential therapeutic peptides, peptidomimetics, or even small molecules that would bind to the HLA-E ligand and abrogate NKG2A/CD94 recognition. Such external intervention would be useful in the emerging field of targeting senescent cells in a variety of age-related diseases, as well as in novel cancer immunotherapies.

## 1 Introduction

**FIGURE 1:**
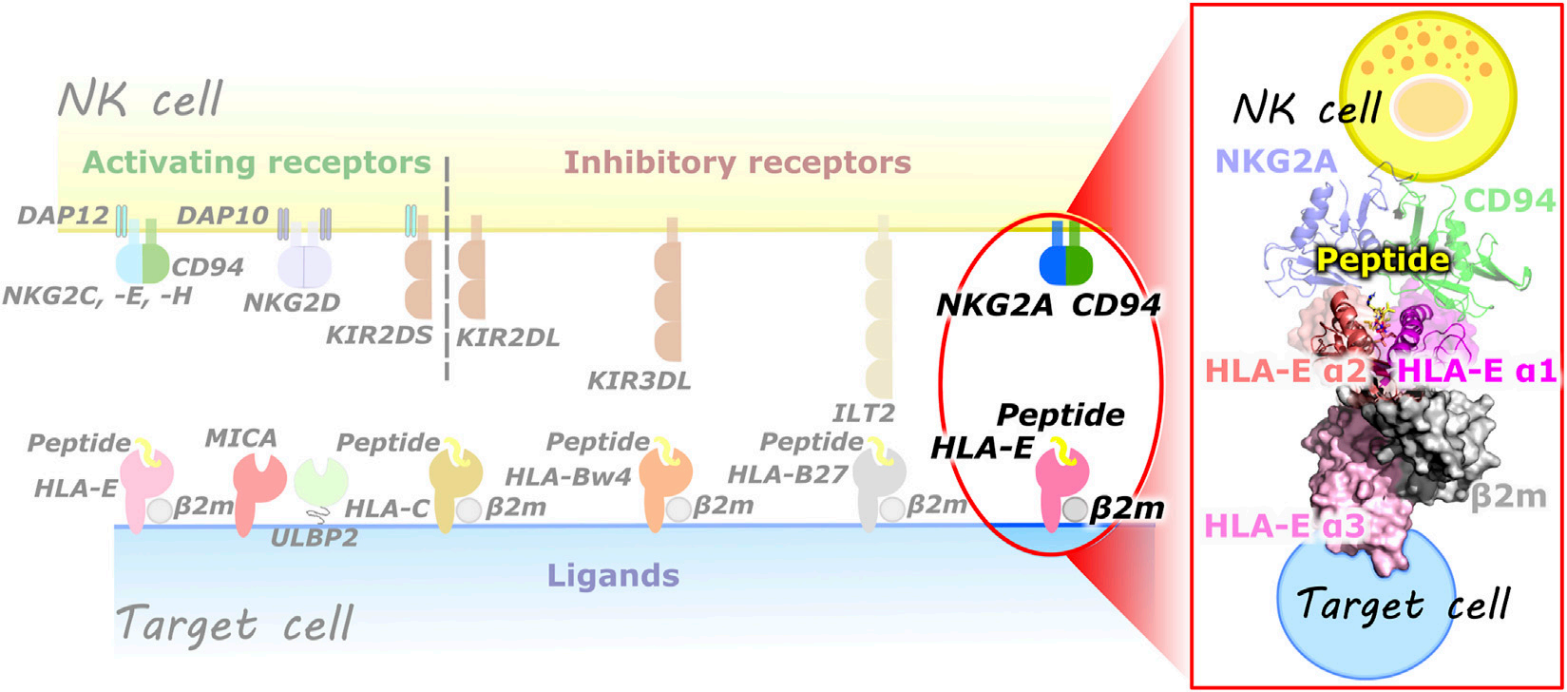
(left) Overview of some of the known NK cell receptors and their corresponding ligands presented on the target cells. (right) Magnified representation of the HLA-E/β2m/peptide/NKG2A/CD94 immune complex comprised of HLA-E heavy chain with three alpha domains (α1–α3), HLA-E light chain β2m, nonameric peptide, CD94 and NKG2A proteins (magenta, salmon, and pink, grey, yellow, green, and blue, respectively).

Natural killer (NK) cells are part of the innate immune system and belong to a lymphocyte lineage with cytotoxic and cytokine-producing functions. They are involved in the organism’s machinery to combat various types of tumors and microbial infections. In addition, they also have regulatory functions as they can influence many other cell types, limiting or enhancing the immune response. NK cells must effectively recognize the target cells while ensuring tolerance to “self” cells (Vivier et al., 2008). This is provided by a variety of activating (e.g., KIR2DS, CD94/NKG2C, -E, -H, NKG2D) and inhibitory (e.g., KIR2DL, KIR3DL, ILT2, CD94/NKG2A, -B) receptors on their cell surface, resulting in a dynamic interplay of antagonistic signals that control the cytotoxic action (Vivier et al., 2008; Borrego et al., 2002; Prašnikar et al., 2021a; Prašnikar et al., 2021b). These receptors are specific for the MHC class I molecules and can be generally divided into three families: 1) killer cell Ig-like receptors, 2) immunoglobulin like transcripts, and 3) C-type lectin receptors. These receptors recognize the classical (class Ia) HLA class I molecules and the non-classical (class Ib) HLA-G and HLA-E molecules. Most representatives of the C-type lectin receptors exist as heterodimers of the CD94 protein covalently linked to a member of the NKG2 family (Borrego et al., 2002) (Figure 1).

Among them the NKG2A/CD94 receptor is one of the inhibitory receptors of the NK and CD8^+^ T-cells that recognizes and acts *via* the HLA-E ligand expressed on the target cells. The HLA-E/NKG2A axis plays a role in colorectal cancer (Eugène et al., 2020), multiple myeloma (Yang et al., 2021), and other cancers (Abd Hamid et al., 2019; Borst et al., 2020) and is rapidly gaining attention as a potentially druggable immune checkpoint. The NKG2A-blocking antibody monalizumab, which targets this axis, has already shown promising results in clinical trials and (Borst et al., 2020) the HLA-E ligand has been recognized as a potential target for the treatment of multiple myeloma (Yang et al., 2021). Overexpression of HLA-E has also been detected in senescent dermal fibroblasts and senescent cells in human skin sections from the elderly, and blocking the HLA-E/NKG2A interaction enhanced the immune response against the senescent cells *in vitro* (Pereira et al., 2019). Thus, the HLA-E/NKG2A axis opens up new promises in many therapeutic areas, and detailed understanding of the key events of molecular recognition is essential for the development of potential therapies.

The HLA-E ligand is expressed on the cell surface as a trimeric complex consisting of the heavy chain, light chain [beta-2 microglobulin (β2m)], and the specific nonameric peptide (further also referred as peptide) derived from the signal sequences of other HLA class I molecules (e.g., HLA-A, -B, -C, -G) (Braud et al., 1998b; Monaco et al., 2008). Remarkably, only specific sequences of the peptide allow high-affinity binding to HLA-E (Braud et al., 1998a; Miller et al., 2003; Ruibal et al., 2020) and its stable surface expression. Moreover, only a handful of them permit effective recognition by the NKG2A/CD94 receptor located on the NK and CD8^+^ T-cells, with the subsequent signal transduction, and resulting target cell protection from elimination (Braud et al., 1998a; Borrego et al., 1998; Valés-Gómez et al., 1999; Michaëlsson et al., 2002; Kaiser et al., 2005; Hoare et al., 2008; Lauterbach et al., 2015a). Furthermore, studies have also shown that not only nonameric peptides, but also longer peptides can bind to HLA-E (Kraemer et al., 2015; Celik et al., 2016). In our previous studies, we proposed this molecular recognition event takes place *via* a tangled network of hydrogen bonds formed in the HLA-E/β2m/peptide/NKG2A/CD94 immune complexes (Prašnikar et al., 2021a).

Some HLA-E-binding nonameric peptides were reported not to elicit protection from the lysis mediated by the NKG2A/CD94^+^ immune cells. These are Hsp60sp, R5V mutated leader peptide of HLA-B7 (Michaëlsson et al., 2002), the leader peptide of HLA-Cw7, and HLA-B27 (Braud et al., 1998a; Borrego et al., 1998; Valés-Gómez et al., 1999; Hoare et al., 2008). There are two possibilities proposed for such a result: 1) the peptide does not provide adequate stabilization of the HLA-E ligand on the surface of the target cell due to its weak binding to it, 2) HLA-E with bound peptide is not actually a ligand for the NKG2A/CD94 receptor. The former has been already shown to be responsible for the lack of protection in cytotoxicity assays for the peptides Cw7 and B27 (Brooks et al., 1999).

Of all the peptides studied so far, the HLA-G leader sequence is the most effective mediator of the inhibitory signaling to the effector immune cells (Kaiser et al., 2005; Lauterbach et al., 2015a) and among the different receptors, the inhibitory CD94/NKG2A receptor is the one with the highest affinity for the HLA-E complex (Lauterbach et al., 2015a). Meanwhile, signal sequences of the HLA-A alleles [VMAPRTL(L/V)L] and the HLA-B7 (VMAPRTVLL) seem to have one of the highest affinities for the HLA-E ligand (Braud et al., 1998a; Hoare et al., 2008).

Available structural and biochemical information on the HLA-E ligand and its bound peptides offer a good starting point for carrying out all-atom molecular simulations in order to unravel the molecular background for their diverse binding affinities (Dror et al., 2012). The acquired data could be then used as the basis for the targeted design of potential therapeutic peptides that would bind to the HLA-E expressed on the target cell surface and abrogate the NKG2A/CD94^+^ immune cell inhibition, over pronounced in some pathological conditions and in senescent cell accumulation. Indeed, therapeutic peptides can represent an attractive alternative to small molecules and proteins having affinities comparable to antibodies and specificities much better than small molecules or antibodies (Shrivastava et al., 2009; Lau and Dunn, 2018). In their development the utilization of computational techniques can play a key role in optimal design of such amino acid-based therapeutics (D’Annessa et al., 2020; Farhadi and Hashemian, 2018).

Here, we report the results of extensive all-atom molecular dynamics (MD) simulations of naturally occurring and alternative designed variants of the nonameric peptides in complex with HLA-E/β2m ligand alone or in the HLA-E/β2m/NKG2A/CD94 complexes to broaden the understanding of the molecular background for their diverse binding affinities which are often associated with only small changes in their structure. Simulations revealed new peptide variants with comparable affinity as natural peptides with highest HLA-E binding affinities. One investigated variant can according to our derived atomistic model (Prašnikar et al., 2021a), interfere with the signal transduction taking place in HLA-E/β2m/peptide/NKG2A/CD94 immune complex thus preventing the target cell from evading the immune clearance *via* NK and CD8^+^ T-cells. With this study, we aim to demonstrate and provide an atomistic picture of how small changes in amino acid sequence can significantly affect such molecular recognition outcomes.

## 2 Materials and Methods

### 2.1 Structural Models

Altogether we built 12 models consisting of HLA-E heavy and HLA-E light chain [called beta-2-microglobulin (β2m)] with various nonameric peptides incorporated into the HLA-E complex along with the apo form of HLA-E. Next, we constructed four models containing the NKG2A/CD94 heterodimer in addition to the HLA-E heavy and light chains and the corresponding nonameric peptide accounting for a total of 17 simulated models. All HLA-E complexes are built using either the crystal structure of the human non-classical major histocompatibility complex molecule HLA-E with bound class Ia MHC leader peptide derived from HLA-B8, solved at 2.85 Å resolution (PDB entry 1MHE) or the crystal structure of human HLA-E, solved at the resolution of 2.50 Å that contains the leader peptide of the HLA class I histocompatibility antigen, alpha chain G (PDB entry 3BZE).

The first model (i) **HLA-E** ^**G_wt**^ consists of HLA-E, β2m, and the G peptide (sequence: VMAPRTLFL), while the remaining models differ in nonameric peptide, incorporated into the HLA-E. The second model (ii) **HLA-E** ^**B27_wt**^ contains a peptide derived from HLA-B27 (sequence: VTAPRTLLL, peptide from PDB entry 1KTL). The rest of the models of the HLA-E complex contain a peptide corresponding to the leader sequence of HLA-B8 and HLA-B7 (sequence: VMAPRTVLL). Namely, the third model (iii) **HLA-E** ^**wt**^ contains the wild type of the B7 peptide. Then, single mutations of the B7 peptide were introduced in the following models: R5V for model (iv) **HLA-E** ^**R5V**^, T6M for (v) **HLA-E** ^**T6M**^, T6A for (vi) **HLA-E** ^**T6A**^, T6F for (vii) **HLA-E** ^**T6F**^, T6Y for (viii) **HLA-E** ^**T6Y**^, T6W for (ix) **HLA-E** ^**T6W**^, T6L for (x) **HLA-E** ^**T6L**^, and T6I for (xi) **HLA-E** ^**T6I**^. We also constructed a model with the wild type B7 peptide with capped peptides’ N-terminal residue, (xii) **HLA-E** ^**cap**^. Capped residues are used to neutralize terminals in peptide chains and for neutralization of peptides’ N-terminal in **HLA-E** ^**cap**^ model we used the ACE (-C(=O)-CH_3_) residue. Finally, we prepared the apo model of HLA-E/β2m, (xiii) **HLA-E** ^**apo**^. In the superscript of all model names the name or the variant of the subjected nonameric peptide is listed.

All simulated HLA-E/β2m/peptide/NKG2A/CD94 complexes are based on the crystal structure of CD94/NKG2A in complex with HLA-E with the incorporated leader sequence of HLA-G, solved at 3.40 Å resolution (PDB entry 3CDG). Peptide G derived from HLA-G, originally present in the crystal structure, was replaced with the peptide B7 (sequence: VMAPRTVLL, peptide from PDB entry 1KPR) to which single mutations were introduced, based on the initial computational assessment of known peptides: T6M in model (xiv) **NKG2A/HLA-E** ^**T6M**^, T6Y in (xv) **NKG2A/HLA-E** ^**T6Y**^, T6W in (xvi) **NKG2A/HLA-E** ^**T6W**^, and T6I in (xvii) **NKG2A/HLA-E** ^**T6I**^. All x-ray structures used in our simulation contain arginine residue at the position 107 thus corresponding to the HLA-E*01:01 variant.

### 2.2 System Preparation and Molecular Dynamics Simulations

For the assignation of protonation states of all ionizable residues PDB2PQR web tool at pH 7 was used (Dolinsky et al., 2004). Carboxylic amino acids were found in their deprotonated states, while histidines were protonated at both imidazole sites or only at Nδ or Nε. The protein complex was embedded in a 10 Å thick layer of TIP3P water molecules (Jorgensen et al., 1983). For the HLA-E complexes, this resulted in a box of 83.144 Å^3^ × 103.623 Å^3^ × 99.798 Å^3^. The assembled systems accommodated approximately 85,000 atoms, including water molecules and 15 Na^+^ counterions. The dimensions of the HLA-E/β2m/peptide/NKG2A/CD94 complexes are 130.968 Å^3^ × 123.440 Å^3^ × 122.079 Å^3^, including water molecules and 18 Na^+^ counterions, the systems numbered approximately 193,500 atoms. Tleap module of Ambertools 18 was used to construct disulfide bonds and model topologies (Case et al., 2018).

First, energy minimization was performed, followed by heating of the systems in two successive steps. In the first step, heating was performed from 0 to 100 K over 5 ps and in the second step from 100 to 303 K over the next 120 ps. During the stepwise heating, positional restraints of 200 kcal/mol Å^2^ and 100 kcal/mol Å^2^ were applied to all heavy atoms in the first and second steps, respectively. Then an isothermal-isobaric ensemble (NPT)-based MD simulation was performed over the next 10 ns, where all restraints were removed and a Berendsen barostat was used to control the pressure of 1 bar (Berendsen et al., 1984). Next, 1.2 μs of MD simulation was carried out using a canonical ensemble (NVT) with periodic boundary conditions and an integration time step of 2 fs, giving a total simulation time of ∼20 µs During the simulation, temperature control (T = 303 K) was ensured with a collision frequency of 1 ps^−1^ using the Langevin thermostat (Loncharich et al., 1992). Hydrogen bonds were constrained using the SHAKE algorithm (Ryckaert et al., 1977) and long-range electrostatic interactions were calculated using the particle mesh Ewald method (Harvey and De Fabritiis, 2009), with a cutoff of 10 Å.

The first ∼200 ns of the NVT MD run were considered an equilibration phase, while the remaining 1 μs of the MD trajectories, stripped of water and counterions, was used for subsequent analysis. MD trajectories were visualized using VMD (Humphrey et al., 1996) and PyMol (SchrӧdingerLLC, 2015) software and analyzed with the *cpptraj* module in Ambertools 18 (Case et al., 2018) and Gromacs 2016 (Van Der Spoel et al., 2005) suite. The cpptraj module in Ambertools 18 (Case et al., 2018) was used for clustering of MD conformations, using a hierarchical agglomerative approach with a distance cutoff of ∼2 Å and a distance metric of mass weighted root-mean-square deviation (RMSD) of backbone atoms (Shenkin and McDonald, 1994).

### 2.3 Binding Free Energy and Interaction Energy Calculations

The binding free energies (ΔG_b_) between the peptide and the rest of the protein complexes as well as its per-residue distribution were calculated by the Molecular Mechanics-Generalized Born Surface Area (MM-GBSA) method (Massova and Kollman, 2000) and the Amber18 code (Case et al., 2018). The calculations were performed using the equilibrated part of the trajectories corresponding to the last 1,000 ns of the MD production run. MM-GBSA calculations were performed for 100 equally spaced frames between 600 and 900 ns of the equilibrated part of the MD trajectory. Per-residue decomposition and energy analyses were performed using a salt concentration of 0.1 M and igb flag value set to 5. In these calculations, the conformational entropic free energy contribution was omitted as it was reported not to contribute to the better quality of the results when using the MM-G(P)BSA (Genheden and Ryde, 2015; Borišek et al., 2020). The interaction energies (ΔE) between the peptide and HLA-E/β2m were additionally calculated using the gmx energy module of the Gromacs 2016 (Van Der Spoel et al., 2005).

### 2.4 Generation of Dynamic Pharmacophores

To gain a deeper understanding of the interaction patterns between different peptide variants and HLA-E ligand, we also derived dynamic pharmacophore models (i.e., dynophores). 1,000 equally spaced frames between 10 ns and 1 μs of the equilibrated parts of the trajectories were exported and analyzed using the DynophoreApp (Bermudez et al., 2015; Sydow, 2015; Bock et al., 2016). The resulting models were then visualized and analyzed in LigandScout (Wolber and Langer, 2005). The LigandScout capabilities were also used to generate apo-site grids of the peptides’ binding pocket on the HLA-E ligand. Buriedness of 0.70, a surface grid of 0.15, and feature probabilities of 1.00 were used for visualization and analysis.

## 3 Results

All models simulated in this study were firmly based on the available crystal structures of the extracellular domains of the human CD94/NKG2A proteins in complex with HLA-E (PDB entry: 3CDG) or HLA-E ligand alone (PDB entry: 3BZE, and 1MHE). Constructed models include all resolved components consisting of extracellular domains of HLA-E, β2m, and nonameric peptide nested between the α1 and α2 domains of the HLA-E, referred to as ligand part. The full immune complexes consisting of ligand and receptor parts also includes the resolved extracellular domains of the NK receptor counterpart, namely proteins NKG2A and CD94 (Figure 1). Together we studied 17 immune complexes (Table 1) of which 13 models consist only of HLA-E ligand part of the immune complex: **HLA-E** ^**G_wt**^, **HLA-E** ^**B27_wt**^, **HLA-E** ^**wt**^, **HLA-E** ^**R5V**^, **HLA-E** ^**T6M**^, **HLA-E** ^**T6A**^, **HLA-E** ^**T6F**^, **HLA-E** ^**T6Y**^, **HLA-E** ^**T6W**^, **HLA-E** ^**T6L**^, **HLA-E** ^**T6I**^, **HLA-E** ^**cap**^, and **HLA-E** ^**apo**^, while the remaining four models also encompass the receptor NKG2A/CD94 counterpart: **NKG2A/HLA-E** ^**T6M**^, **NKG2A/HLA-E** ^**T6Y**^, **NKG2A/HLA-E** ^**T6W**^, and **NKG2A/HLA-E** ^**T6I**^, where the superscript indicates the name or the variant of the subjected nonameric peptide.

**TABLE 1:** List of constructed models of the HLA-E and HLA-E/NKG2A complexes with specific names of the model, nonameric peptide presence in the complex, its sequence, the mutations introduced, and the crystal structure used to build the model on.

	Model	PDB entry	Peptide	Peptide sequence	Introduced mutation of the peptide
HLA-E complex	**HLA-E** ^**G_wt**^	3BZE	G	VMAPRTLFL	—
	**HLA-E** ^**B27_wt**^	1MHE	B27	VTAPRTLLL	—
	**HLA-E** ^**wt**^	1MHE	B7	VMAPRTVLL	—
	**HLA-E** ^**R5V**^	1MHE	B7	VMAP**V**TVLL	R5V
	**HLA-E** ^**T6M**^	1MHE	B7	VMAPR**M**VLL	T6M
	**HLA-E** ^**T6A**^	1MHE	B7	VMAPR**A**VLL	T6A
	**HLA-E** ^**T6F**^	1MHE	B7	VMAPR**F**VLL	T6F
	**HLA-E** ^**T6Y**^	1MHE	B7	VMAPR**Y**VLL	T6Y
	**HLA-E** ^**T6W**^	1MHE	B7	VMAPR**W**VLL	T6W
	**HLA-E** ^**T6L**^	1MHE	B7	VMAPR**L**VLL	T6L
	**HLA-E** ^**T6I**^	1MHE	B7	VMAPR**I**VLL	T6I
	**HLA-E** ^**cap**^	1MHE	B7	V^**cap**^MAPRTVLL	capping
	**HLA-E** ^**apo**^	1MHE	—	—	—
NKG2A/HLA-E complex	**NKG2A/HLA-E** ^**T6M**^	3CDG	B7	VMAPR**M**VLL	T6M
	**NKG2A/HLA-E** ^**T6Y**^	3CDG	B7	VMAPR**Y**VLL	T6Y
	**NKG2A/HLA-E** ^**T6W**^	3CDG	B7	VMAPR**W**VLL	T6W
	**NKG2A/HLA-E** ^**T6I**^	3CDG	B7	VMAPR**I**VLL	T6I

Bold marked amino acids in Peptide sequence denote the mutated residue.

### 3.1 Identification of the Prospective Peptide Mutation Points by the End-Point Binding Free Energy Calculations

**FIGURE 2:**
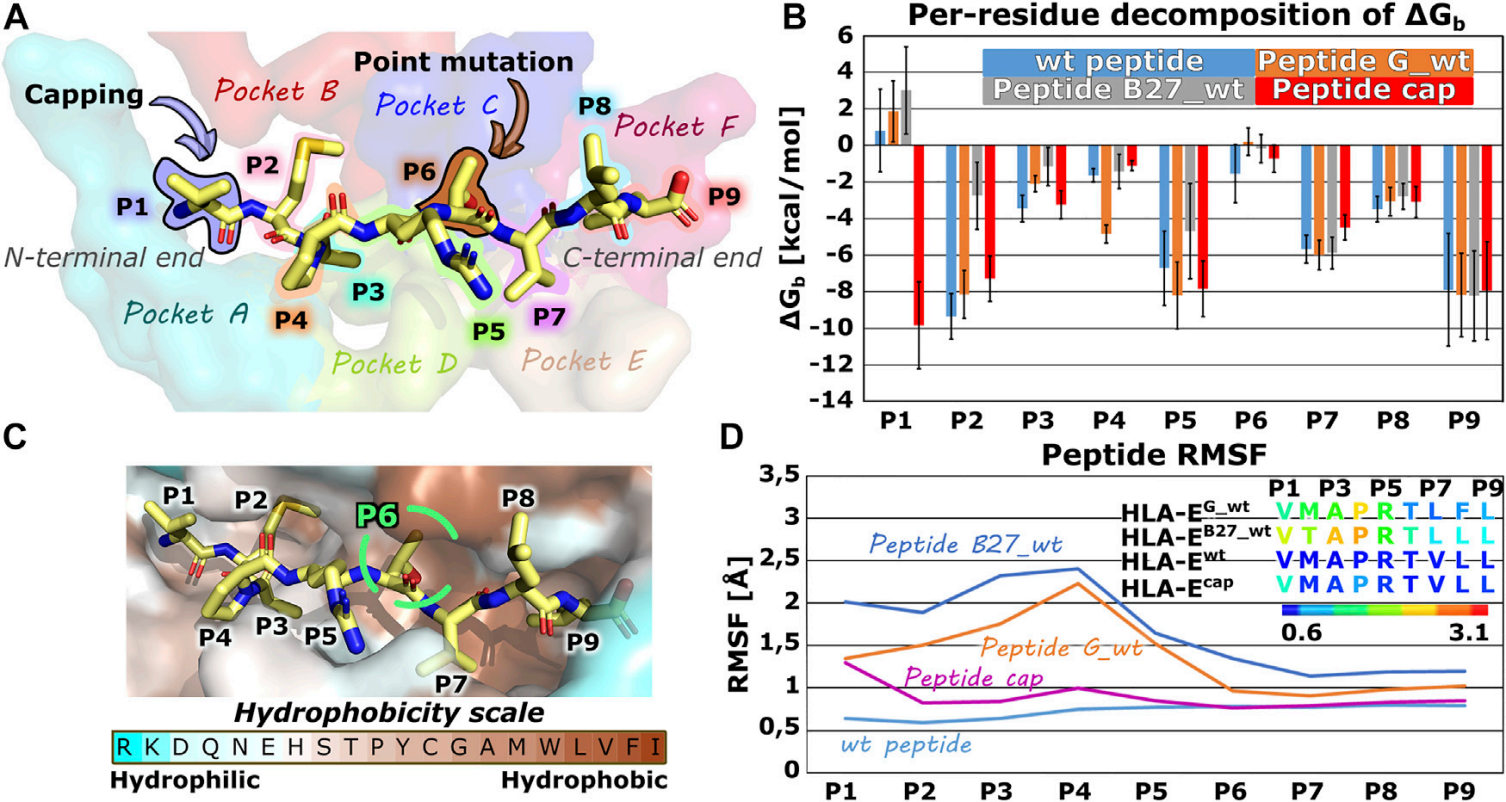
Nonameric peptide residue positions, binding pockets, altering points, binding site, energetic profile and per-residues Root mean square fluctuation (RMSF) values. **(A)** Wild type peptide B7 with highlighted residue positions P1-P9 and alteration points with binding pockets on the HLA-E/β2m accommodating peptides’ side chains. **(B)** Per-residue decomposition of the mean MM-GBSA binding free energy calculated over 100 equally spaced frames between 600 and 900 ns of the equilibrated part of the MD trajectories for HLA-E/β2m models including wild type peptides **B7** (blue), **G** (orange), and **B27** (grey) and capped peptide **cap** (red). **(C)** B7 peptide in its HLA-E binding pocket, colored according to the amino acid hydrophobicity (Sweet and Eisenberg, 1983). **(D)** Per-residues Root mean square fluctuation (RMSF) values calculated on the equilibrated part of the Molecular Dynamics (MD) trajectories for wild type peptides **B7** (light blue), **G** (orange), and **B27** (grey) and capped peptide **cap** (violet) for residues’ Cα atoms. Marked whiskers denote the obtained standard deviation.

In the first part of the study, we evaluated the structural features and dynamic behavior of the HLA-E ligand part of the immune complex by simulating three different peptides (peptide G, B27, and B7) with experimentally reported affinities for HLA-E (Braud et al., 1998a). Per-residue decomposition of the binding free energy of the simulated models **HLA-E** ^**wt**^, **HLA-E** ^**G_wt**^, and **HLA-E** ^**B27_wt**^ using the MM-GBSA method (Genheden and Ryde, 2015; Borišek et al., 2020) (Supplementary Table S1; Figure 2) revealed the binding contribution of each peptide residue and provided the rationale for the selection of the mutation points. In agreement with the literature, B7 and G peptides’ positions 2 and 9 represent the energetically most important anchor points. This has been demonstrated experimentally by mutation studies (Braud et al., 1997; Miller et al., 2003), the conservation of amino acids in naturally occurring HLA-E binding peptides (Ruibal et al., 2020), and solved crystal structures. In the latter, deep and shallow binding pockets were observed to accommodate the side chains of P2, P9, as well as P7, and P3 in addition to P6, respectively (Supplementary Table S2) (O’Callaghan et al., 1998). Moreover, in case of the peptide B27 the anchor role of P2 is weakened upon replacement of Met with Thr which is in line with previous reports of Thr not being a preferred amino acid at this position (Miller et al., 2003). Per-residue decomposition additionally revealed that positions 5 and 7 also make a substantial contribution to binding, while the contribution of amino acids at positions 1, 4, and 6 was significantly smaller (Figure 2).

Proline residue at position 4 is conserved in naturally occurring peptides derived from the class Ia HLA signal sequences (O’Callaghan et al., 1998; Chen et al., 2014), which may well indicate an important structural function *via* an imposed conformational constraint, so its mutation could significantly affect binding and was thus not considered. Hence, to develop a B7 peptide variant with higher affinity for HLA-E ligand, peptide interactions at positions 1 or 6, besides P4 proline, could be optimized as these residues of the peptide have the smallest per-residue binding free energy contributions.

Interestingly, position 1 represents the N-terminal residue of the peptide and its positive charge could be the main reason for its poor contribution to binding observed in the energetic analysis (Figure 2). To test this initial hypothesis that the charge of the N-terminal residue negatively affects the interactions of P1 with the binding pocket in HLA-E/β2m ligand, we decided to cap the N-terminal amino acid with ACE (-C(=O)-CH_3_) capping residue, and perform another MD simulation. Comparison of the per-residue decompositions of the capped peptide of model **HLA-E** ^**cap**^ with the wild type peptide **HLA-E** ^**wt**^ (Supplementary Table S1; Figure 2) corroborated the positive charge of the N-terminus as a likely culprit for the antagonistic contribution of P1 to peptide binding.

Furthermore, capping indeed enhanced the interactions of P1 residue in **HLA-E** ^**cap**^ with its surroundings to the extent that it can be considered as one of the anchoring residues. In contrast, the RMSF calculation showed that capping of the P1 slightly destabilized the binding of the peptide at the N-terminal site, particularly the residue P1 as it now became more flexible. However, compared with the bound peptides G and B27 in **HLA-E** ^**G_wt**^ and **HLA-E** ^**B27_wt**^, the capped peptide still exhibits an overall low flexibility (Figure 2; Supplementary Table S3).

**FIGURE 3:**
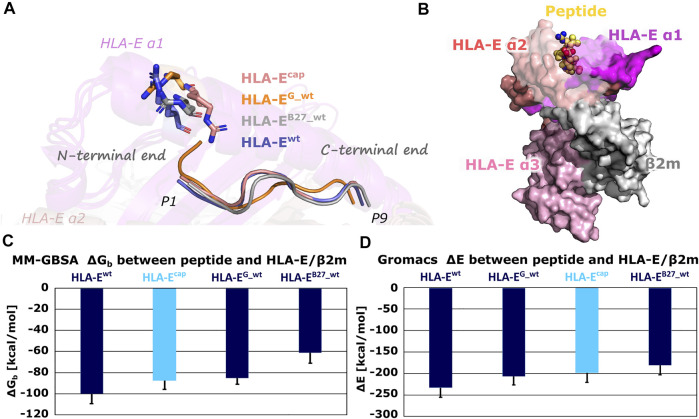
**(A)** Conformations of the Arg62^HLA-E^ residue in most representative clusters of **HLA-E** ^**cap**^, **HLA-E** ^**G_wt**^, **HLA-E** ^**B27_wt**^, and **HLA-E** ^**wt**^. Compared to other models, the Arg62^HLA-E^ residue points more towards C-terminal in the **HLA-E** ^**cap**^ model. **(B)** B7 peptide in yellow depicted as spheres and HLA-E (magenta, slate and pink)/β2m (gray) ligand complex **HLA-E** ^**wt**^ in surface representation. **(C)** Determined mean binding free energies (ΔG_b_) between peptides and HLA-E/β2m calculated by MM-GBSA and **(D)** interaction energies (ΔE) between peptides and HLA-E/β2m calculated with Gromacs. Marked whiskers denote the obtained standard deviation.

Subsequent comparison of the most representative clusters extracted from the MD trajectories (the last 1 μs of simulation time) revealed an interesting orientation of the side chain of the Arg62^HLA-E^ residue in **HLA-E** ^**cap**^ model. Namely, in **HLA-E** ^**wt**^, **HLA-E** ^**G_wt**^, **HLA-E** ^**B27_wt**^ models containing wild-type peptides, Arg62^HLA-E^ residue is extending toward the N-terminal site of the peptide, while in the **HLA-E** ^**cap**^ model its side chain was pointing more upwards extending in the direction of the C-terminal site of the peptide (Figure 3). This observation is in line with a similar experimentally determined orientation that was described in the MHC-I complex with N-terminally extended, N-methylated 10-mer peptide (Li et al., 2019). Such positioning of Arg62^HLA-E^ was proposed to open pocket A of the peptide binding site, allowing elongated peptides to protrude from the binding groove (Li et al., 2019). Thus, based on our results it could be suspected that this distinct conformation of Arg62^HLA-E^ might be the consequence of the capping.

The MM-GBSA binding free energy as well as Gromacs interaction energy calculations successfully reproduced the experimentally determined order of binding affinities of all three wild-type peptides B7, G, and B27 for HLA-E/β2m (Supplementary Table S4; Figure 3) (Braud et al., 1998a). This provides the necessary reassurance that these calculations can be used to generally distinguish between the peptides with higher or lower affinity for the HLA-E. Furthermore, binding free energy calculations for the **HLA-E** ^**cap**^ model revealed that capping of P1 did not improve the binding affinity of the peptide. Both, binding free energy and interaction energy between the capped peptide and the HLA-E/β2m ligand proved to be less favorable than the energies of the **HLA-E** ^**wt**^ with bound B7 peptide and closer to that of the **HLA-E** ^**G _wt**^ model.

These results suggest that the nonameric peptide’s P6 position holds the most potential for further optimization to enhance its binding to the HLA-E ligand. Finally, we analyzed the peptide binding site on the HLA-E with the grid apo-site analysis performed in Ligandscout (Wolber and Langer, 2005) as another viable approach to search for possible favorable interaction areas in this binding pocket. This analysis, which is unbiased in the respect to the existing bound ligands, revealed positive ionizable, aromatic, and hydrophobic pharmacophore features in close proximity to the P6 site further substantiating the previous analysis (Supplementary Figure S1).

### 3.2 Mutation of the 6th Position of the Nonameric Peptide

Based on the results obtained, the hydrophobic nature seems to be strongest near the P6 site, where the introduction of additional charged structural elements could lead to a desolvation penalty (Salari and Chong, 2010), which could be problematic for peptide binding. This implies that the introduction of hydrophobic residues I, F, L, W, M, A, Y at this position could be considered good substitutions for more optimal binding.

**FIGURE 4:**
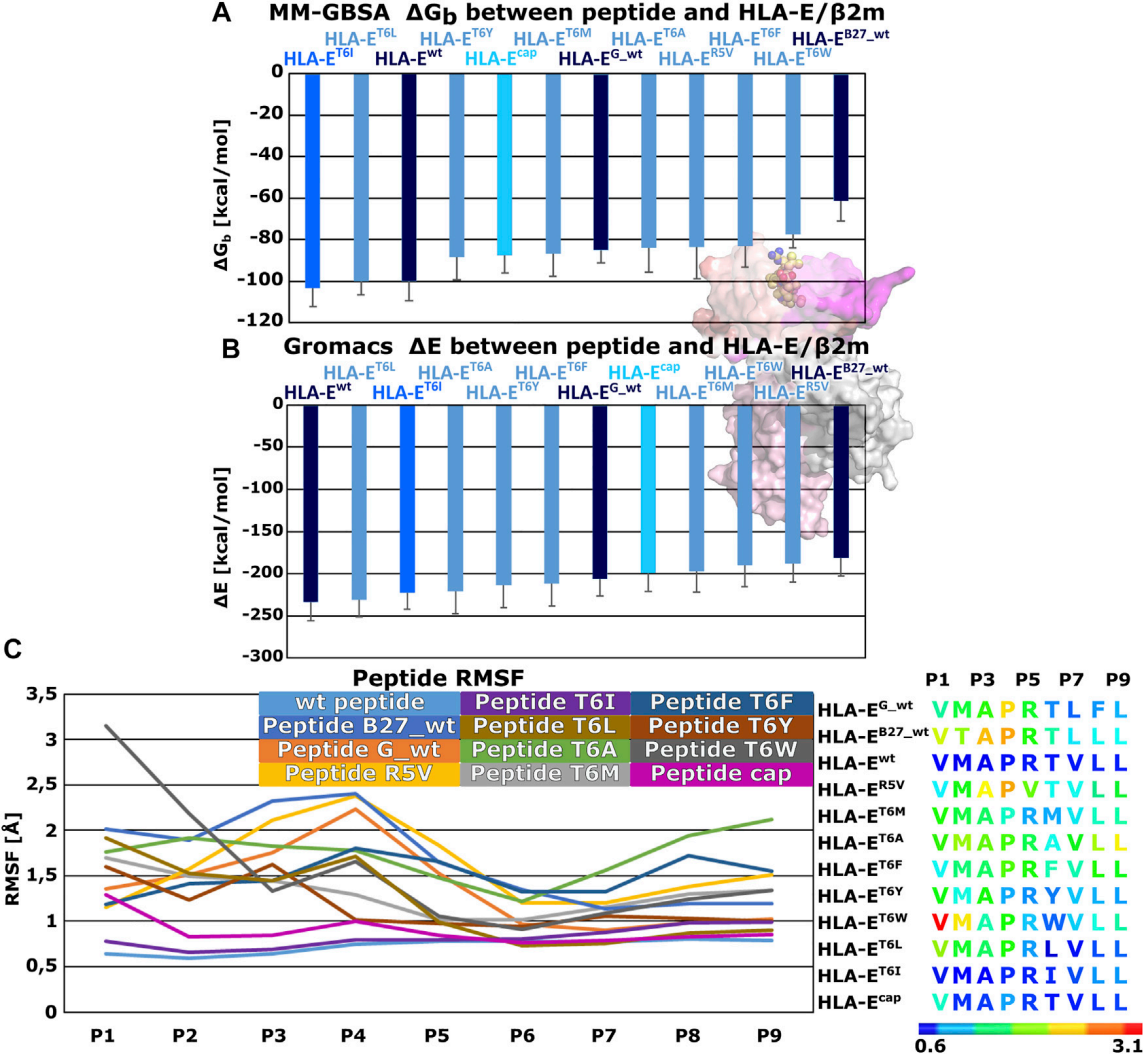
Binding free energy calculations between peptides and HLA-E/β2m and Per-residues Root mean square fluctuations (RMSF). **(A)** Mean binding free energies (ΔG_b_) calculated by MM-GBSA method. **(B)** mean interaction energies (ΔE) calculated with Gromacs. **(C)** Per-residues Root mean square fluctuation (RMSF) values calculated on the equilibrated part of Molecular Dynamics (MD) trajectories for peptides: wild type peptide G (**G_wt**), B27 (**B27_wt**), B7 (**wt**), B7 variants **R5V**, **T6I**, **T6L**, **T6A**, **T6M**, **T6F**, **T6Y**, **T6W**, and B7 (**cap**) (left panel). Sequences of subjected peptides in corresponding models colored according to RMSF values from blue to red indicating increased flexibility (right panel). Marked whiskers denote the obtained standard deviation.

Therefore, we generated these seven variants of the peptide B7 that differ in the amino acid at P6 position to evaluate the effects of this point mutation on the peptides binding to HLA-E ligand. Additionally, we also simulated another model, where we introduced the R5V mutation to the B7 peptide, which has been shown to abrogate the cytolytic effect of NKG2A/CD94^+^ NK cells (Michaëlsson et al., 2002) in order to assess R5V peptide binding properties. Determined MM-GBSA binding free energies and Gromacs interaction energies both showed that designed peptide variant T6I in the **HLA-E** ^**T6I**^ system has a comparable affinity to the initial wild type peptide B7 in **HLA-E** ^**wt**^. Both energy calculations also suggested good affinity for designed peptide T6L in **HLA-E** ^**T6L**^ (Supplementary Table S4; Figure 4). Subsequent RMSF calculations assessing the peptides flexibility revealed that of all the simulated peptides, the B7 wild type peptide was the most stably bound in the binding pocket (Figure 4; Supplementary Table S3). Further comparison of the RMSFs of the wild-type peptides shows that the peptides are most stably bound at the C-terminal site of the peptides. This can also be observed for all mutant peptides. Moreover, peptide T6I is similarly stable in its binding pocket as the peptide B7, whereas the same is not true for the peptide T6L. Less flexibility of the peptide may imply stronger binding, so these observations further corroborate the observed comparable binding affinities of peptides B7 and T6I. Although the B7 peptide shows higher flexibility at the N-terminal site after capping, the capped peptide still shows low overall flexibility compared to peptides other than the T6I-B7 variant, being in line with the trend observed in the models involving wild type peptides.

**FIGURE 5:**
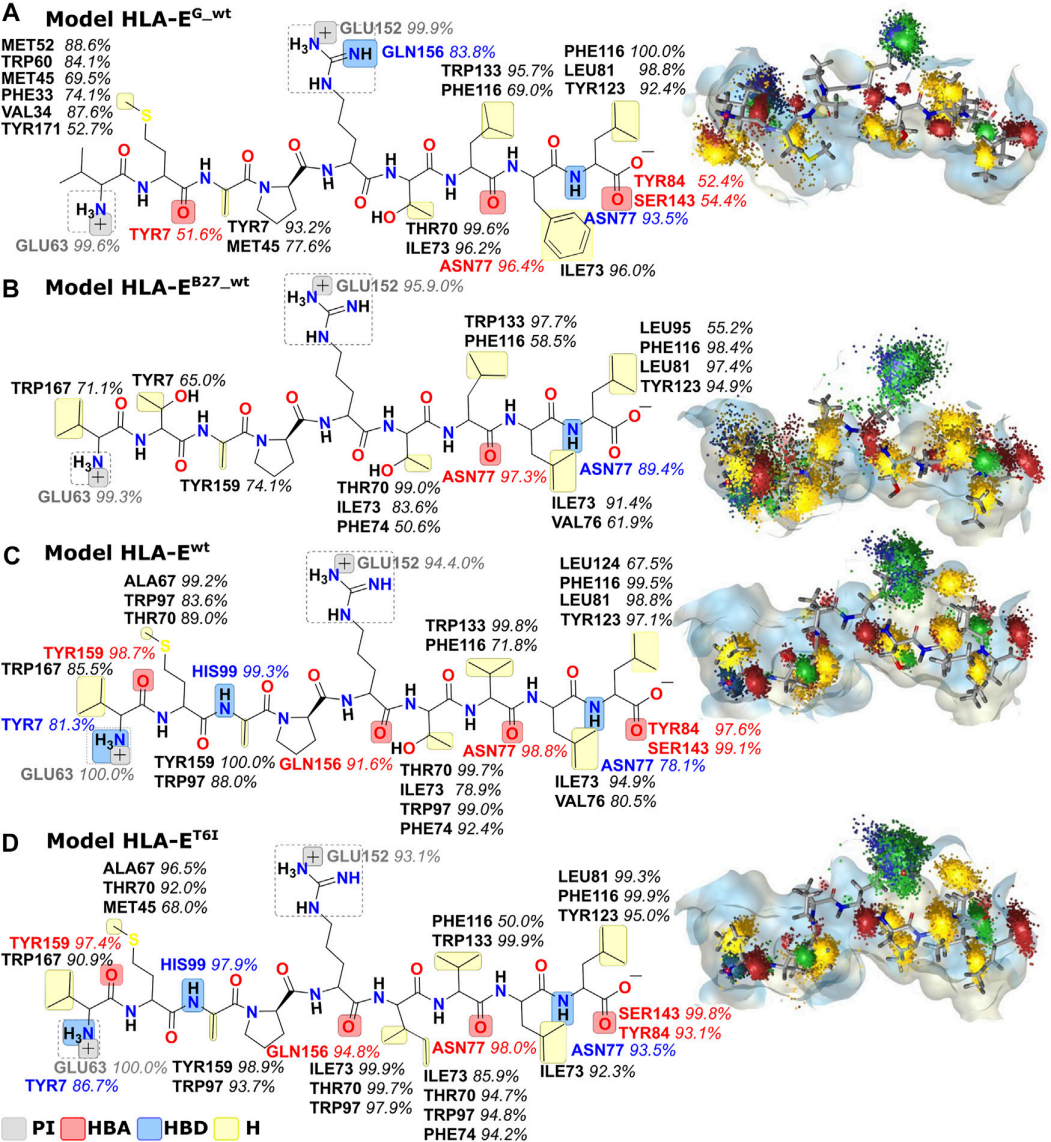
Dynophore models for the wild type peptide models **(A) HLA-E** ^**G_wt**^, **(B) HLA-E** ^**B27_wt**^, and **(C) HLA-E** ^**wt**^ and B7 peptide variant **(D) HLA-E** ^**T6I**^ calculated for 1,000 uniformly distributed frames between 10 ns and 1 μs of the last 1 μs of MD trajectories. Only interactions that occur in at least 50% of the trajectory are mapped. From the visualization on the right, it is evident that peptides with better binding affinities have more condensed interactions. PI, positive ionizable; HBA, hydrogen bond acceptor; HBD, hydrogen bond donor; H, hydrophobic area.

**FIGURE 6:**
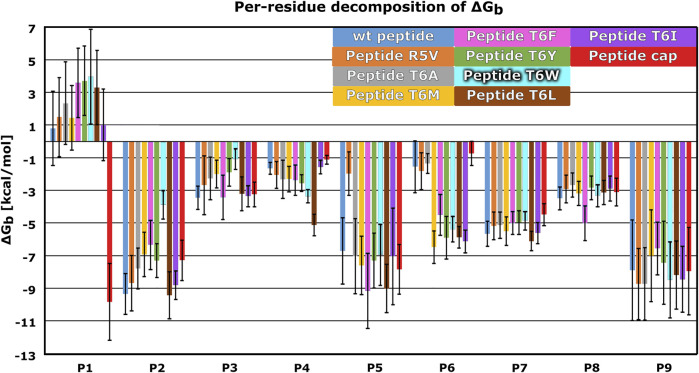
Mean per-residue decomposition of MM-GBSA Binding Free Energies (ΔG_b_) calculated over 100 equally spaced frames between 600 and 900 ns of the last 1 μs of the MD trajectories for HLA-E/β2m models including variants of the B7 peptides: wild type (**wt)** (blue), **R5V** (orange), **T6A** (grey), **T6M** (yellow), **T6F** (pink), **T6Y** (green), **T6W** (cyan), **T6L** (brown), **T6I** (violet), and capped B7 (**cap**) (red). Marked whiskers denote the obtained standard deviation.

Next, we created dynamic 3D pharmacophore (dynophore) models, also known as dynamic pharmacophores, for each of the 12 simulated HLA-E:peptide complexes. In them, dynamic nature of the MD simulations and static structure-based pharmacophore models are combined, resulting in a dynamical characterization of the peptides interaction patterns throughout the simulations (Janežič et al., 2021). In this way, we have added another layer of analysis to the previously described energy-based investigation by now focusing more on the geometric aspect of molecular recognition between the peptides and HLA-E ligand with included dynamical component provided by the MD simulations. According to the dynophore analysis, peptides B7 in **HLA-E** ^**wt**^ and B7_T6I mutant peptide in **HLA-E** ^**T6I**^ system form similar interactions with HLA-E with also comparable intensities. The obtained dynophores also demonstrated that in models containing wild-type peptides with lower affinity for HLA-E than peptide B7, namely peptides G in **HLA-E** ^**G_wt**^ and B27 in **HLA-E** ^**B27_wt**^, the pharmacophore features are more scattered than in the **HLA-E** ^**wt**^ model, where they were observed to be more condensed (Figure 5). We also observed that isoleucine on P6 in the **HLA-E** ^**T6I**^ system forms additional hydrophobic interactions with Trp97 while its branched side chain enables extra contact points with Ile73, Thr70, and Trp97 residues. Moreover, per-residue decomposition of the binding free energy by the MM-GBSA method confirmed that the contribution of the 6I residue of B7_T6I to binding in **HLA-E** ^**T6I**^ system is indeed greater than the contribution of T6 residue of the B7 peptide (Figure 6).

We further substantiated the dynophore geometric analysis with per-residue MM-GBSA decomposition analysis for the generated peptide variants and compared it to the observations obtained for the three wild type peptides with experimentally reported data. All mutations at the peptides 6th residue, with the exception of T6A, resulted in greater contribution of P6 to the binding (Figures 2, 6; Supplementary Figures S2, S3). However, the point mutations in most of these cases did not result in the overall improved peptide:HLA-E/β2m binding (Supplementary Table S4, Figure 4).

The per-residue analysis further shows that in all simulated cases the C-terminal site of the peptide is more stably bound, as here two residues (P7 and P9) with large contribution to binding could be observed. On the other hand, only one such residue (P2) could be found at the peptide N-terminal site. The central residue (P5) also makes a large contribution to binding (Figures 2, 4, 6; Supplementary Tables S1, S3). Joining our observations from dynophore, MM-GBSA, and RMSF analyses together, we can detect three anchor residues, namely P2, P7, and P9 which is in line with the behavior observed for the wild type peptides. Considering only B7 variants, with exclusion of the peptide variant T6A, P6 could be considered as another anchor residue as binding contributions were substantially improved compared to P6 of the wild type peptides. This confirms that the selection of amino acids that were introduced into P6 can improve the interaction to this part of the peptide binding pocket. It should be noted that P5 also serves as a residue with a significant contribution to peptide binding and is not accommodated in any of the HLA-E binding pockets but protrudes from the binding furrow and is thus putatively involved in recognition by CD94/NKG2A receptor.

In agreement with previous reports (Ruibal et al., 2020), our simulations have confirmed that P6 is indeed intolerant for the incorporation of bulky amino acids, as P6 mutation to W in the **HLA-E** ^**T6W**^ system resulted in an early detachment of the N-terminal site (residues P1–P4) of the peptide from its binding pocket. Interestingly, the C-terminal site of the peptide (residues P5–P9) remained bound until the end of this simulation (Supplementary Figure S4). Additionally to the model **HLA-E** ^**T6W**^, minor detachments of the peptides at the N-terminal side were observed also in models **HLA-E** ^**G_wt**^, **HLA-E** ^**T6L**^, **HLA-E** ^**T6Y**^ **, HLA-E** ^**T6M**^, and **HLA-E** ^**T6W**^. In them only the P1 residue was protruding from the binding pocket, while P2 moves from HLA-E pocket B towards pocket A (Supplementary Table S2). While minor differences in the peptides’ N-terminal side positioning in the binding groove were observed, no major conformational differences in the simulated models were noticed (Supplementary Figures S5–S8). We also detected that in most representative clusters of HLA-E models containing B7 variants, Arg62^HLA-E^ was similarly aligned as in the wild-type models, further confirming the role of capping of the N-terminal amino acid for the orientation of Arg62^HLA-E^ toward the C-terminal peptide site discussed above (Supplementary Figure S9). These results advocate that binding of the peptide might be further improved by securing better interactions at the N-terminal site, thereby preventing the observed detachments, leading to a more active contribution of the N-terminal site to the HLA-E binding.

### 3.3 Assessment of NKG2A/CD94 Interaction With the Ligand Variants

**FIGURE 7:**
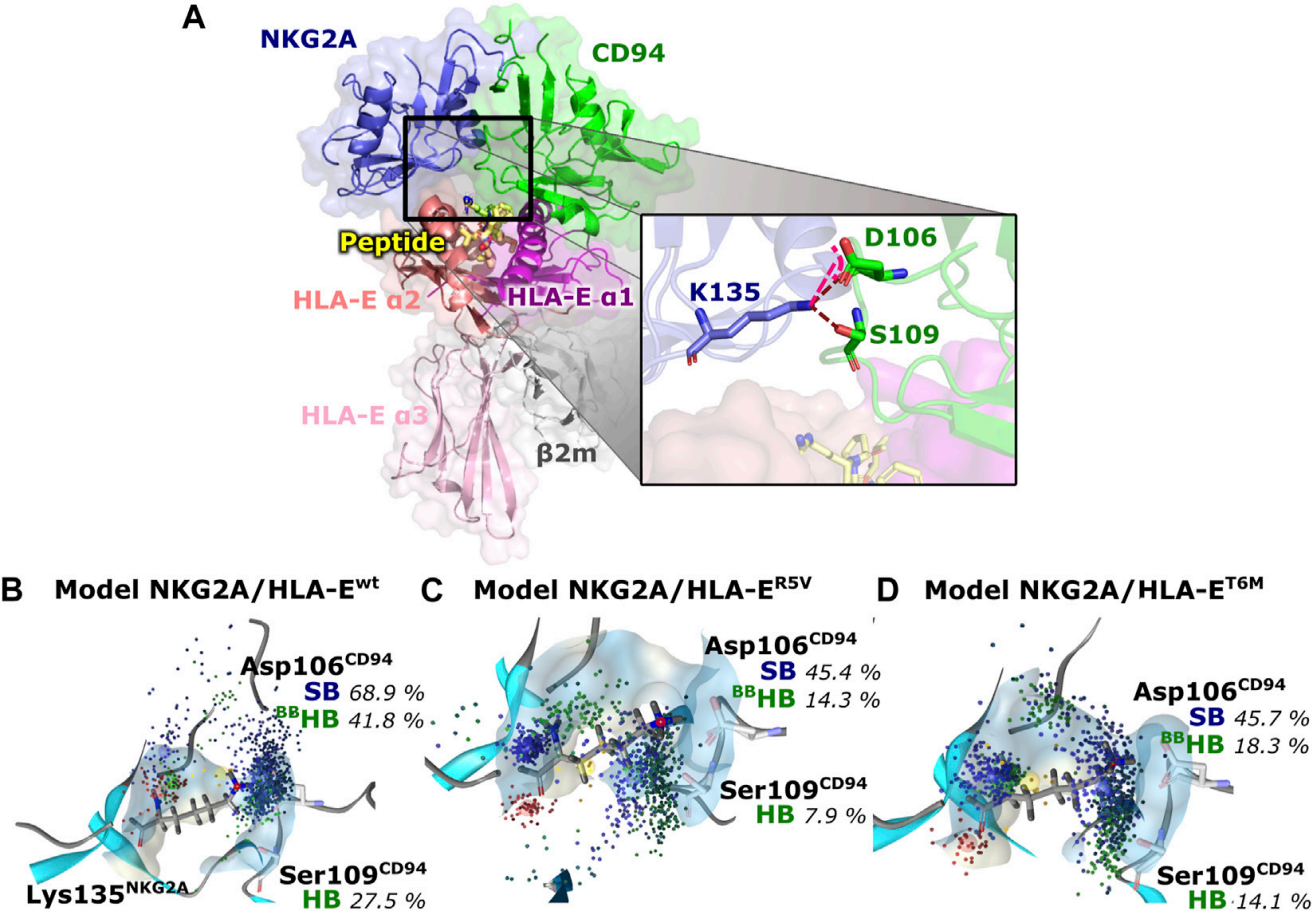
Representation of the full immune complex and dynophores for Lys135^NKG2A^ interactions. **(A)** Full immune complex involving ligand HLA-E (α1 (magenta), α2 (salmon) and α3 (pink) domains)/β2m (grey)/peptide (yellow) and receptor NKG2A (blue)/CD94 (green) with magnified key residues Lys135^NKG2A^, Asp106^CD94^, and Ser109^CD94^ and illustrated salt bridge (pink) and hydrogen bonds (brown) putatively involved in inhibitory signal transduction (Prašnikar et al., 2021a). **(B)** Dynophore for Lys135^NKG2A^ of the immune complexes **NKG2A/HLA-E** ^**wt**^, **(C) NKG2A/HLA-E** ^**R5V**^, and **(D) NKG2A/HLA-E** ^**T6M**^ for 1,000 uniformly distributed frames between 10 ns and 1 μs of the equilibrated part of the trajectories. The percentage of trajectories in which an interaction is present is given for each model. More condensed interactions correspond to the more stable interaction. SB, salt bridge; HB, hydrogen bond; ^BB^—the interaction is formed with the oxygen backbone atom of Asp106^CD94^.

Finally, we evaluated whether the introduced point mutations in the nonameric peptide can lead to the abrogation of the interaction between HLA-E and inhibitory receptor NKG2A/CD94, resulting in the inhibition of the cytotoxicity of the effector immune cells. To this end, we examined the influence of several designed peptide variants on the occurrence and stability of the hydrogen bond between the Lys135^NKG2A^ and Ser106^CD94^ and on the salt bridge between Lys135^NKG2A^ and Ser109^CD94^ formed between receptor proteins. In our previous work, these interactions were shown to play key roles in ensuring inhibitory signal transduction (Figure 7; Supplementary Figure S10) (Prašnikar et al., 2021a).

Together, we simulated and analyzed four HLA-E/β2m/peptide/NKG2A/CD94 immune complexes with designed variants of the peptide B7 containing the following mutations: T6M, T6Y, T6W, and T6I. The T6I variant was selected because of its previously discussed favorable HLA-E binding properties. Because the T6W variant partially detached from the HLA-E binding pocket in the **HLA-E** ^**T6W**^ model, we wanted to further test whether this behavior would lead to the disruption of the proposed signal transduction mechanism. The remaining two simulated peptide variants with mutations T6Y or T6M were selected to expand the “chemical space” studied. Namely, in addition to their general hydrophobic nature, both introduced amino acids, contain functional groups in their side chains that distinguish them from other point mutations. This could lead to different results in terms of the final receptor (NKG2A/CD94) and ligand (HLA-E/β2m/peptide) recognition and outcome.

In addition to the variants of peptide B7 mutated at position 6, we also present here some new data that we extracted from the simulated HLA-E/β2m/peptide/NKG2A/CD94 complexes with peptides G, B7, B7 with R5V mutation, and its apo model, that we generated and simulated in the scope of our previous work where we studied the molecular recognition events necessary for the inhibitory signal transduction (Prašnikar et al., 2021a). According to the literature, peptides B7 and G enable inhibition of cytotoxic activity (Valés-Gómez et al., 1999). In contrast, the R5V mutation of B7 has been reported to completely abrogate the HLA-E-mediated protection against killing by the CD94/NKG2A-expressing NK cells (Michaëlsson et al., 2002).

Measurements of the Asp106^CD94^-Lys135^NKG2A^ and Ser109^CD94^-Lys135^NKG2A^ interaction distances as well as their assessment *via* dynophore models led to the conclusion that T6Y, T6W, and T6I peptide variants are likely to allow the receptor-ligand recognition to take place, thereby preventing the cytotoxic action of the NK cell. On the other hand, the **NKG2A/HLA-E** ^**T6M**^ model with T6M mutation of the B7 peptide shows the presence of the proposed key interactions at frequencies closer to the negative **NKG2A/HLA-E** ^**R5V**^ model containing the B7_R5V peptide that abrogates HLA-E-mediated protection. Furthermore, the **NKG2A/HLA-E** ^**T6M**^ model also has similar average distances between the heavy atoms of the interacting residues as **NKG2A/HLA-E** ^**R5V**^. In contrast, the remaining three models with petide variants display comparable features to the models that allow inhibitory signal transduction: **NKG2A/HLA-E** ^**G_wt**^ and **NKG2A/HLA-E** ^**wt**^ (Prašnikar et al., 2021a) with bound peptides G and B7 with respect to the monitored three key interactions (Table 2; Figure 7; Supplementary Figure S10).

**TABLE 2:** Average distances between key atoms and percentages of trajectory in which interaction (SB, salt bridge; HB, hydrogen bond) is present.

Interaction	Asp106^CD94^@CG-Lys135^NKG2A^@NZ		Asp106^CD94^@O-Lys135^NKG2A^@NZ		Ser109^CD94^@OG-Lys135^NKG2A^@NZ	
Model	Avg. Dist. [Å]	SB, trajectory percentage	Avg. Dist. [Å]	HB, trajectory percentage	Avg. Dist. [Å]	HB, trajectory percentage
NKG2A/HLA-E^G_wt^	5.1	67.5	4.3	40.4	4.0	37.9
NKG2A/HLA-E^wt^	5.1	68.9	4.5	41.8	5.5	27.5
NKG2A/HLA-E^R5V^	6.0	45.4	6.0	14.3	6.6	7.9
NKG2A/HLA-E^T6M^	6.1	45.7	5.7	18.3	6.1	14.1
NKG2A/HLA-E^T6Y^	5.4	52.3	4.3	40.9	4.7	29.2
NKG2A/HLA-E^T6W^	5.8	57.7	4.8	27.3	4.2	30.0
NKG2A/HLA-E^T6I^	4.6	74.2	3.9	52.0	4.4	25.7
NKG2A/HLA-E^apo^	6.2	40.5	5.7	16.4	6.3	9.1

## 4 Discussion

The MHC class I molecules bind short endogenous peptides during their initial folding in the endoplasmic reticulum, providing the mechanism to monitor abnormal protein production (Jensen et al., 1999). Peptides are generated with the proteasome, transported in the endoplasmic reticulum by an MHC-encoded heterodimeric member of the ATP-binding cassette family of transporters (TAP), the peptide is further cleaved by the endoplasmic reticulum aminopeptidase (ERAP), and loaded in the peptide loading complex to HLA-E (Jensen et al., 1999; Blees et al., 2017; Ogg et al., 2019). HLA-E binds almost exclusively nonameric peptides derived from the signal sequences of MHC Ib molecules, but binding of alternative peptides has also been observed after TAP or ERAP disruption (Ogg et al., 2019).

**FIGURE 8:**
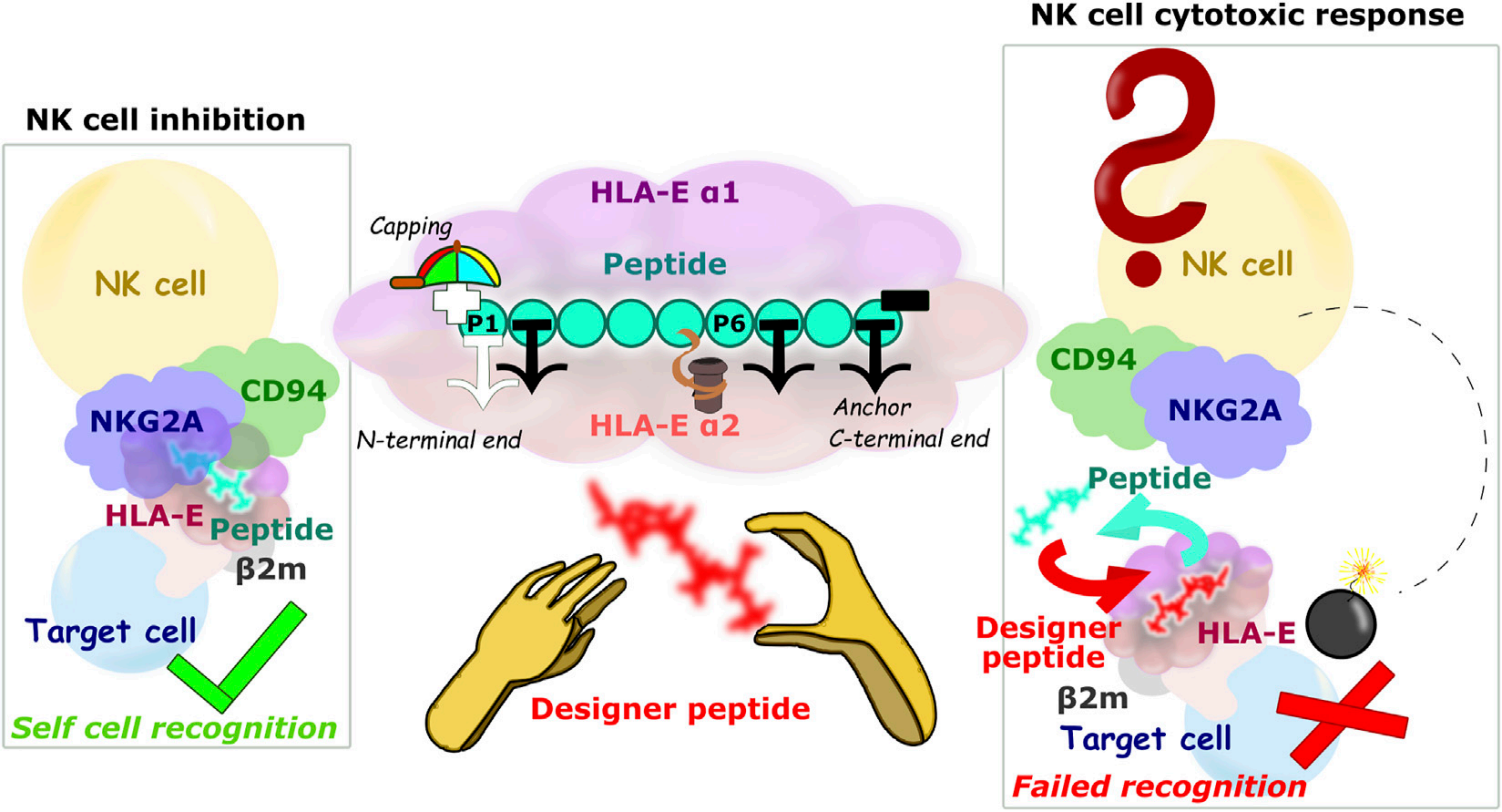
Schematic representation of the HLA-E/β2m/peptide/NKG2A/CD94 axis of the immune complex studies. (left) Inhibition of NK cell-mediated cytotoxicity through the harmonious network of interactions between different parts of ligand and receptor, (center) Main study results: 1) the P2, P7, and P9 anchor positions, 2) significant contribution of the P5 residue to binding, 3) securing the P1 anchor with N-terminal capping of the B7 peptide, and 4) introduction of hydrophobic amino acid can improve the binding contribution of P6, nonetheless introduction of bulky amino acid on P6 can result in peptides’ detachment from the binding furrow. (right) Hypothetical scenario in case the designer peptide is introduced and abolishes the interaction between the HLA-E ligand and the NKG2A/CD94 receptor.

It has been reported that for strong binding to the HLA-E, it is preferred that the nonameric peptide consists of methionine on position 2 and isoleucine or leucine on position 9 (Michaëlsson et al., 2002). Additionally positions 6, 7 and 3 were also shown to have a marked impact on binding affinity and thereby HLA-E cell-surface stabilization. Together they were labeled as anchor residues (Miller et al., 2003; Hoare et al., 2008). Also, the relevance of position 5 is repeatedly reported, as Arg is preferred on this position (Michaëlsson et al., 2002; Miller et al., 2003; Petrie et al., 2008). In fact, amino acids on peptide positions 5 and 8 presumably serve as a major contact residues to communicate with the CD94/NKG2 receptor on the immune system natural killer (NK) cells and CD8^+^ T-cells (Miller et al., 2003; Hoare et al., 2008). According to recent work main anchor positions 2 and 9 prefer large hydrophobic residues, while position 7 can accommodate large hydrophobic, small or rigid residues. Lastly, positions 1, 3, and 6 seem to hold preference for smaller residues as this might promote binding of anchor residues at positions 2 and 7 (Figure 8) (Ruibal et al., 2020).

By performing an extensive computational study of molecular recognition between wild type peptides G, B27 and B7 with known HLA-E binding properties we identified two peptide positions with the least contribution to binding, namely P1 and P6. We further explained that the negative contribution of P1 to peptide binding is likely a consequence of the N-terminal charge, since capping of P1 resulted in a favorable energy contribution of P1 to peptide binding. Therefore, changing P1s -NH_3_^+^ to -CH_3_ could lead to more optimal interactions between P1 and HLA-E. This retrospective section of our study provided the essential data of the peptides binding, serving as the baseline to assess further peptide alterations.

By generating and simulating several variants of the B7 peptide we demonstrated the preference of position 6 for isoleucine, as the T6I mutation resulted in the highest binding affinity. On the other hand, we confirmed that P6 is indeed incompatible for bulky amino acids as previously suggested (Ruibal et al., 2020). Namely, the T6W mutation resulted in the peptide detachment at the N-terminal site, indicating that tryptophan interferes most with binding of the P2 anchor position and also reduces the contribution of the P3 residue, but apparently has no effect on the interactions of the C-terminal anchors P7 and P9. From this and the calculated per-residue distribution of binding free energy, of all peptides, we can infer that the N-terminal site (P1–P4) is more weakly bound to the binding furrow of the peptide than the C-terminal site (P6–P9) of the peptide. This prompt us with notion that optimizing peptide binding at the N-terminal site would be more prudent to ensure better binding. Peptide processing with ERAP offers a possible explanation for the observed bindings, as this enzyme trims the MHC-I bound precursor peptides at their N-terminal sites, allegedly after peptides bind to its HLA-E binding pocket with their C-terminal residues (Li et al., 2019). However, the stronger binding at the C-terminal site could also be an indication that it is more important for receptor interaction. Thus, mutations in this part might be more likely to lead to impaired interaction with HLA-E receptors.

Although the T6M peptide variant does not result in enhanced binding to HLA-E in the **HLA-E** ^**T6M**^ compared to the wild type peptide B7, the simulation of the **NKG2A/HLA-E** ^**T6M**^ model predicts that this variant is likely to abolish recognition and inhibitory signal transduction *via* the inhibitory receptor CD94/NKG2A when bound to HLA-E. The observed key interactions mimicked the behavior of the model with the R5V peptide variant that abrogates HLA-E-mediated protection. As in our previous work (Prašnikar et al., 2021a) no large-scale conformational changes were observed while simulating HLA-E/β2m/peptide ligand or full HLA-E/β2m/peptide/NKG2A/CD94 immune complexes. This further substantiates the notion how a small change in amino acid sequence can lead to a profound effect on binding and molecular recognition in this important biological system.

Some support of this observation could be found in the experiments performed with the HLA-B leader peptides. The HLA-B leader peptides with methionine on the P2 position (e.g., peptide B7) binds to HLA-E with much higher affinity as those with threonine (e.g., peptide B27) (Braud et al., 1998a) and this was also confirmed in our computational study (see Section 3.1). Peptide binding also secures the HLA-E expression on the cell surface, and consequently HLA-B leader peptides with methionine on P2 result in high and those with threonine on P2 in moderate level of the surface HLA-E ligand (Lauterbach et al., 2015b). In addition, HLA-E surface density is also affected by the *01:01, *01:03 HLA-E polymorphism (Lauterbach et al., 2015b).

Nonetheless, higher peptide affinity for HLA-E does not lead to stronger inhibition of the effector immune cell cytotoxic effect as HLA-G leader sequence (peptide G) is the most effective mediator of the inhibitory signalling (Kaiser et al., 2005; Lauterbach et al., 2015b), despite having relatively low affinity for HLA-E (Braud et al., 1998a) and low surface expression (Lauterbach et al., 2015b). Furthermore, in our previous work, we simulated the HLA-B27 leader peptide in full immune complex in which it displayed features closer to the models that do not allow inhibitory signal transduction *via* NKG2A/CD94 (Prašnikar et al., 2021a). Thus, the HLA-B27 leader peptide in complex with HLA-E might indeed not be a ligand for the NKG2A/CD94 receptor. Moreover, it could be possible that either the HLA-B leader peptide-dependent HLA-E surface density or the ability of the HLA-B leader peptide to mediate signals through the inhibitory receptor NKG2A/CD94 could influence the (re)education and responsiveness of donor immune effector cells (Watzl et al., 2014; He and Tian, 2017) in hematopoietic stem cell transplantation and lead to different outcomes depending on the polymorphism in the exon 1 of HLA-B (Petersdorf et al., 2020a; Petersdorf et al., 2020b).

In this study we show that MD simulations can be used as tools to predict the peptide binding affinity for HLA-E and the potential of the resulting immune complex to inhibit NK or CD8^+^ T-cell-mediated cytolysis *via* the NKG2A/CD94 interaction. The data obtained also lay the necessary structure-based foundation for future development of potential therapeutic peptides, peptidomimetics, or even small molecules that would bind to the HLA-E ligand and then abrogate NKG2A/CD94 recognition. The utility of any dynamic model obtained can be further extended to describe the nature of peptide binding to HLA-E/β2m at the atomistic level, offsetting the disadvantage of the lower throughput of MD simulations compared with cell assays (D’Annessa et al., 2020). It should be noted that our previous studies of molecular recognition between HLA-E ligand and either NKG2A/CD94 or NKG2C/CD94 receptors outlined different key residues and ligand receptor interactions as being vital for signal transduction (Prašnikar et al., 2021a; Prašnikar et al., 2021b). Hence, the results obtained by utilizing the NKG2A/CD94 receptor cannot be straightforwardly transferred to other HLA-E receptors such as NKG2C or T-cell receptor (TCR).

Designer peptides, which are likely the next step in the development towards potential therapeutic application of the outlined approach, could theoretically bind to HLA-E intracellularly or extracellularly. Since MHC class I molecules generally do not bind internalized peptides, but only those formed within the cell (Jensen et al., 1999), designer peptides for intracellular incorporation into the HLA-E complex would likely need to be introduced into the cell in a form of vaccine that allows their intracellular assembly (vector, mRNA vaccines). However, this fact results in several challenges that would have to be overcome, such as selective uptake, TAP permissibility for uptake of designer peptide to the endoplasmic reticulum, and the influence of peptide loading complex machinery on designer peptide binding. In cellular immunology, peptides are commonly added to cell cultures to test the NK and T-cell responses, and this results in antigen specific activation. There are several processes that can mediate this presentation, including cross-presentation (Adiko et al., 2015; Embgenbroich and Burgdorf, 2018; Montealegre and Van Endert, 2019), which allows the presentation of internalized peptides on the MHC-I molecules such as HLA-E.

On the other hand, extracellularly, designer peptides could bind to the HLA-E/β2m as a “rescue peptide” (Chang et al., 2011; Walters et al., 2020) after dissociation of the peptide originally presented by HLA-E/β2m on the cell surface (Jensen et al., 1999; Kraemer et al., 2015; Sturm et al., 2020). Lower pH values could be beneficial for such peptide exchange, as they would facilitate dissociation of the original peptides (Jensen et al., 1999; Sturm et al., 2020). Indeed, mild acid elution is one of the most commonly used methods to secure the cellular MHC peptide ligands (Sturm et al., 2020). In fact, a lower pH was detected at tumor sites (Pillai et al., 2019) and in inflamed tissues (Menkin and Warner, 1937; Punnia-Moorthy, 1987), which is characteristic of the environment of senescent cells (Prašnikar et al., 2020). This circumstance could favorably complement the upregulation of HLA-E on these cells (Pereira et al., 2019; Eugène et al., 2020; Yang et al., 2021) and provide the necessary selectivity in the exchange of the original with designer peptide in case of therapeutic application. Furthermore, if such designer peptide in complex with HLA-E is not a ligand for the inhibitory receptor, NKG2A/CD94^+^ immune cells would be able to remove the target cells previously protected with HLA-E/β2m/peptide (Figure 8).

To conclude, our extensive molecular dynamics (MD) simulations of the HLA-E/β2m/peptide complex enhanced the atomistic understanding of the peptide binding to its binding pocket on HLA-E and the influence of single mutations to binding. Subsequent simulations of HLA-E/β2m/peptide/NKG2A/CD94 immune complex shed a light on the effect of introduced point mutations on the inhibitory signal transduction that protects the cell from the cytotoxic effects of the immune NK and CD8^+^ T-cells. Furthermore, performed simulations provide an atomistic interpretation of how a small change in the amino acid sequence can lead to a profound effect on binding and molecular recognition ultimately effecting the signal transduction. Subsequent experimental corroboration would be possible *via* cytotoxicity assays using NKG2A^+^ NK cells as effector cells and HLA-E-expressing cells previously incubated with proposed peptides as target cells. In them caspase-3 activity and CD107a expression levels could be monitored to detect the NK cell cytotoxicity and activity. Knowledge provided under the scope of this study can form the basis for the targeted development of designer peptides that would bind to the HLA-E expressed on the target cell surface and abrogate the NKG2A/CD94^+^ immune cell inhibition, over pronounced in some pathological conditions and in senescent cell accumulation, potentially leading to new cancer immunotherapies as well as new treatment of age-related diseases, by modulating the number of senescent cells.

## Data Availability

The raw data supporting the conclusion of this article will be made available by the authors, without undue reservation.
