# Supplementary material for: What a Difference an Amino Acid Makes: An All-Atom Simulation Study of Nonameric Peptides in Inhibitory HLA-E/NKG2A/CD94 Immune Complexes
Source: Front Pharmacol. 2022 Aug 4;13:925427. doi: 10.3389/fphar.2022.925427 (PMC9385950; doi:10.3389/fphar.2022.925427)
Supplement: Supplementary file 1 [file DataSheet1.pdf]

Supplementary Material

What a Difference an Amino Acid Makes: An All-Atom Simulation Study of Nonameric Peptides in Inhibitory HLA-E/NKG2A/CD94 Immune Complexes

Eva Prašnikar[1,2], Andrej Perdih[3,4*], Jure Borišek[1*]

[1]Laboratory for chemical informatics, National Institute of Chemistry, Theory department, Ljubljana, Slovenia

[2]Graduate School of Biomedicine, University of Ljubljana, Faculty of Medicine, Ljubljana, Slovenia

[3]Laboratory for Computational Biochemistry and Drug Design, National Institute of Chemistry, Theory department, Ljubljana, Slovenia

[4]Faculty of Pharmacy, University of Ljubljana, Ljubljana, Slovenia

*** Correspondence:**
Andrej Perdih: andrej.perdih@ki.si
Jure Borišek: jure.borisek@ki.si

1 Supplementary Figures and Tables

1.1 Supplementary Figures

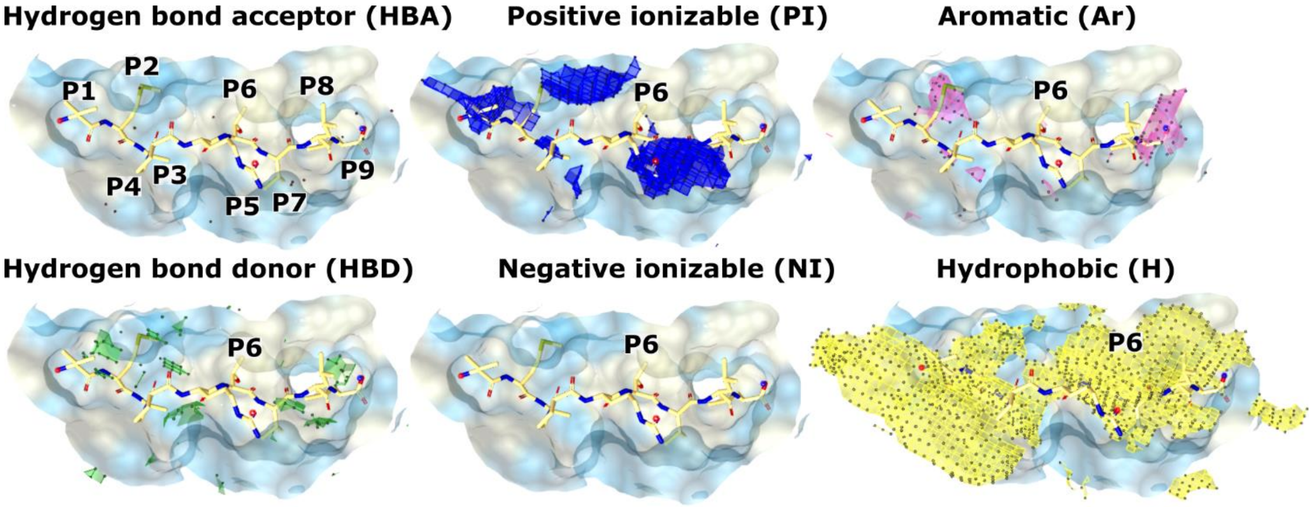

Supplementary Figure 1. Apo site grid (Wolber and Langer, 2005) of the peptide binding pocket in the HLA-E/β2m protein complex. Apo site grids were obtained using LigandScout applying the following parameters: buriedness 0.70, surface grid 0.15, feature probabilities 1.00. Each pharmacophoric feature (Hydrogen bond acceptor (red)/donor (green), positive (blue)/negative (red) inonizable, aromatic (pink), hydrophobic (yellow)) is represented in separate representation of the binding pocket.

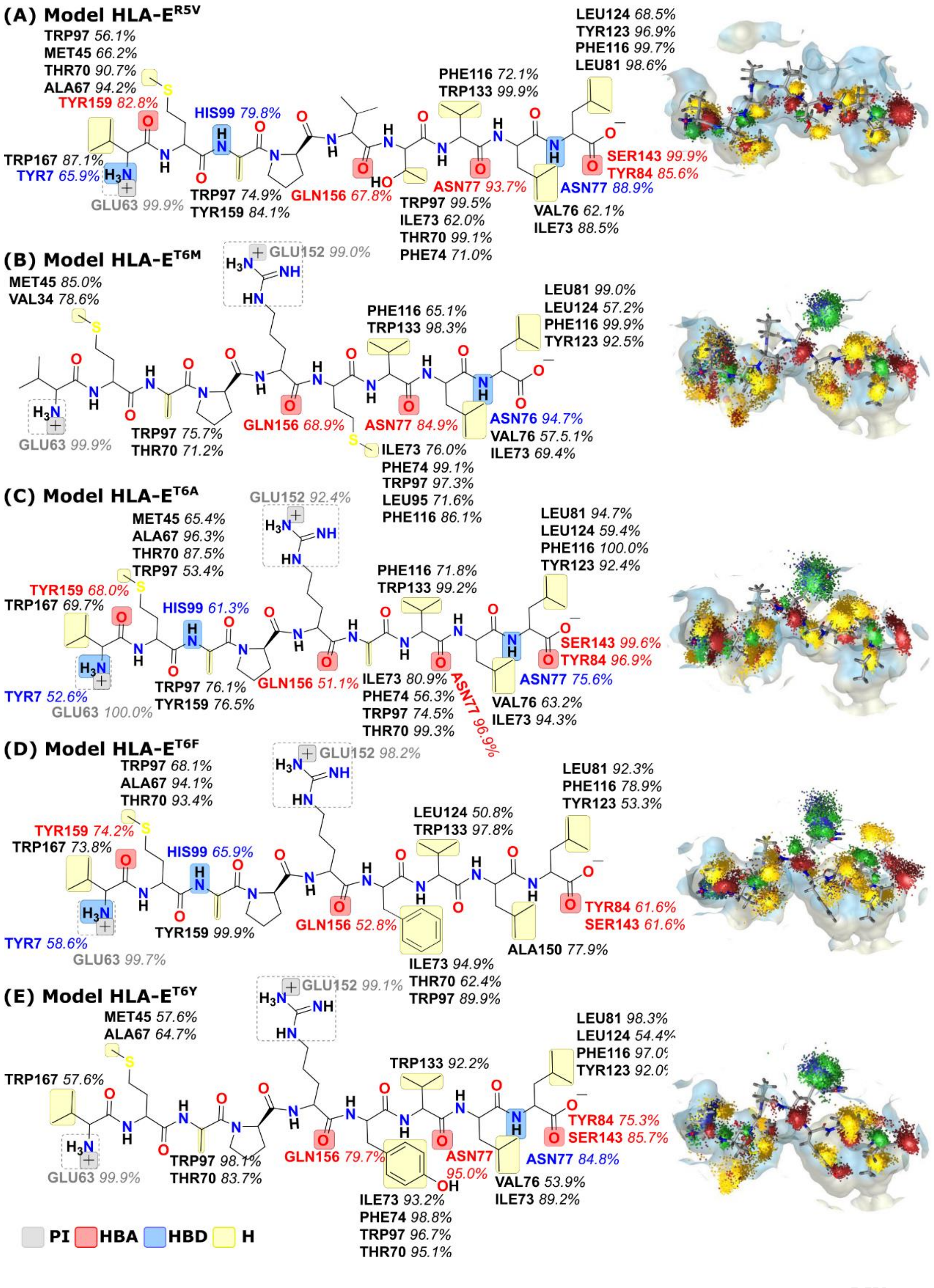

Supplementary Figure 2. Dynophores for B7 peptide variants models (**A**) **HLA-E^{R5V}**, (**B**) **HLA-E^{T6M}**, (**C**) **HLA-E^{T6A}**, (**D**) **HLA-E^{T6F}**, and (**E**) **HLA-E^{T6Y}** calculated for 1000 uniformly distributed frames between 10 ns and 1 μs of simulation time on equilibrated parts of the trajectories. Only interactions that occur in at least 50% of the trajectory are mapped. From the visualization on the right, it is evident that peptides with better binding affinities have more condensed interactions. PI – positive ionizable, HBA – hydrogen bond acceptor, HBD – hydrogen bond donor, H – hydrophobic area.

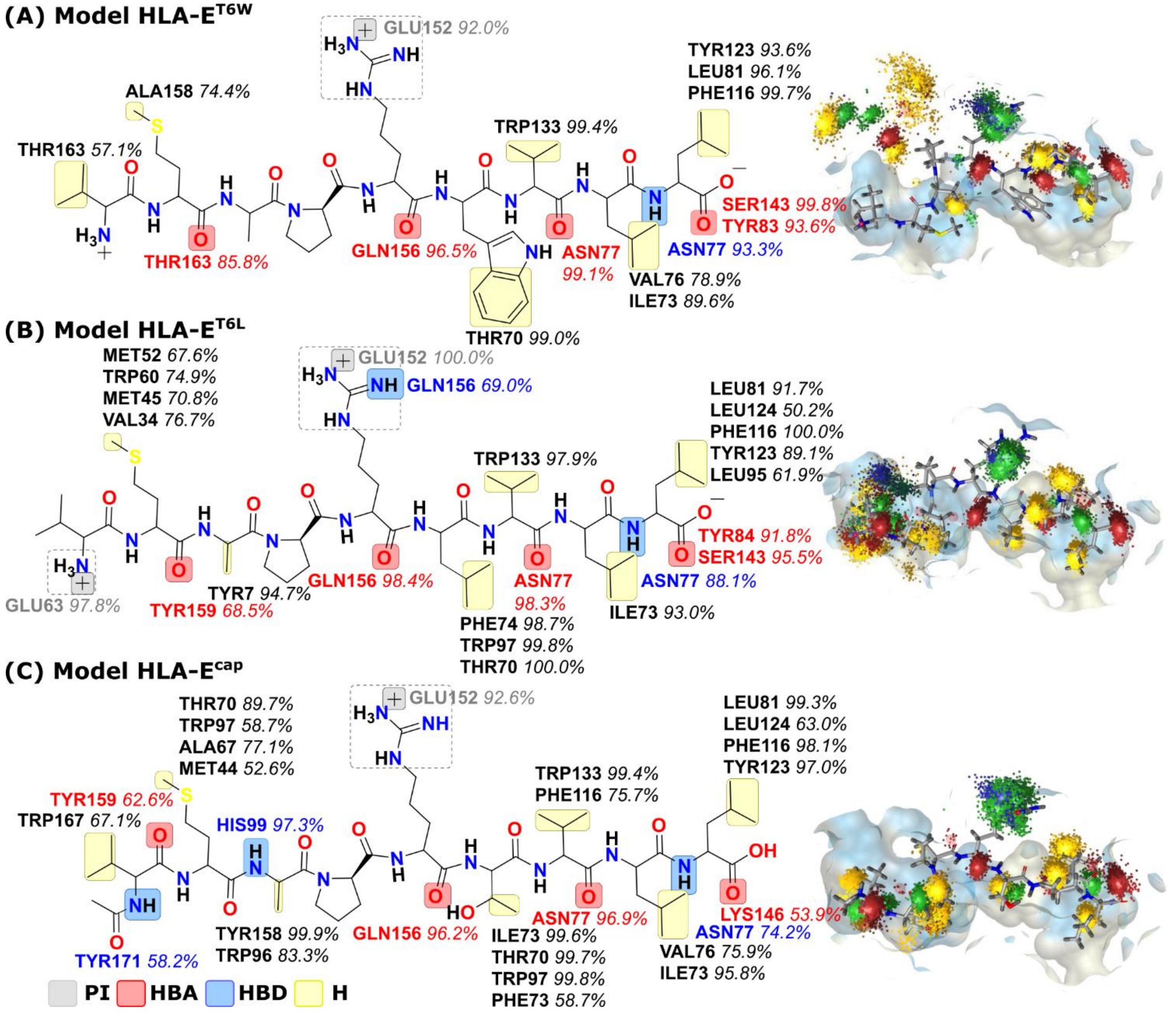

Supplementary Figure 3. Dynoohores for B7 peptide variants models (**A**) **HLA-E^{T6W}**, (**B**) **HLA-E^{T6L}**, and (**C**) **HLA-E$^{B7–cap}$** for 1000 uniformly distributed frames between 10 ns and 1 µs of simulation time on equilibrated parts of the trajectories. Only interactions that occur in at least 50% of the trajectory are mapped. From the visualization on the right, it is evident that peptides with better binding affinities have more condensed interactions. PI – positive ionizable, HBA – hydrogen bond acceptor, HBD – hydrogen bond donor, H – hydrophobic area.

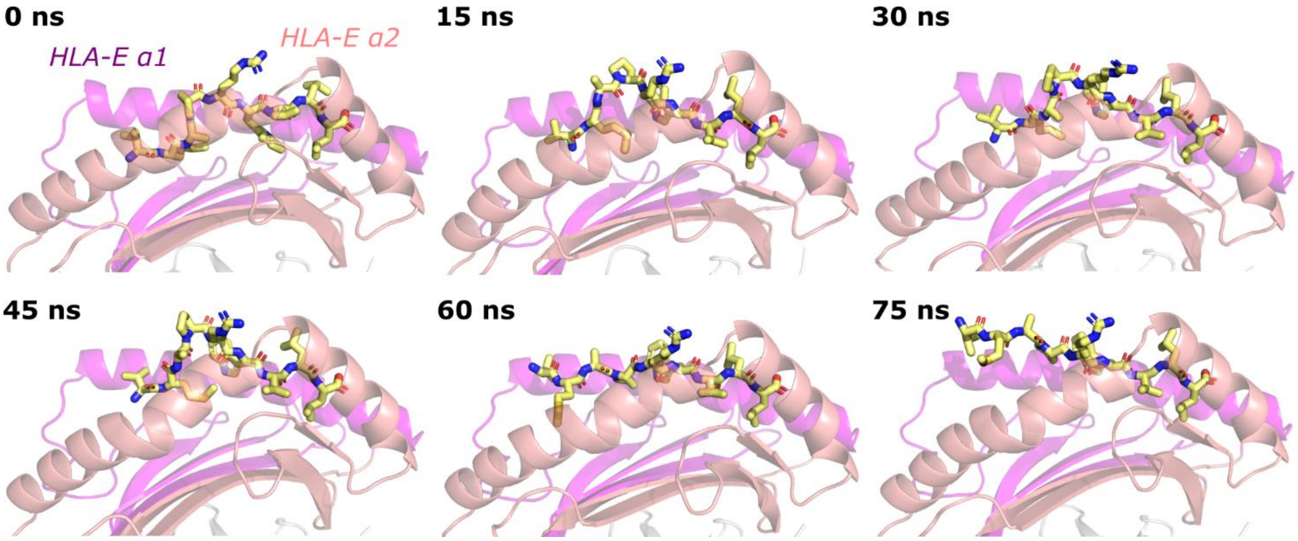

Supplementary Figure 4. Snapshots of the equilibrated MD trajectory (last 1 μs of simulation time) for model **HLA-E$^{\mathbf{T6W}}$** at selected time points. Full peptides' N-terminal detachment can be seen at 75 ns.

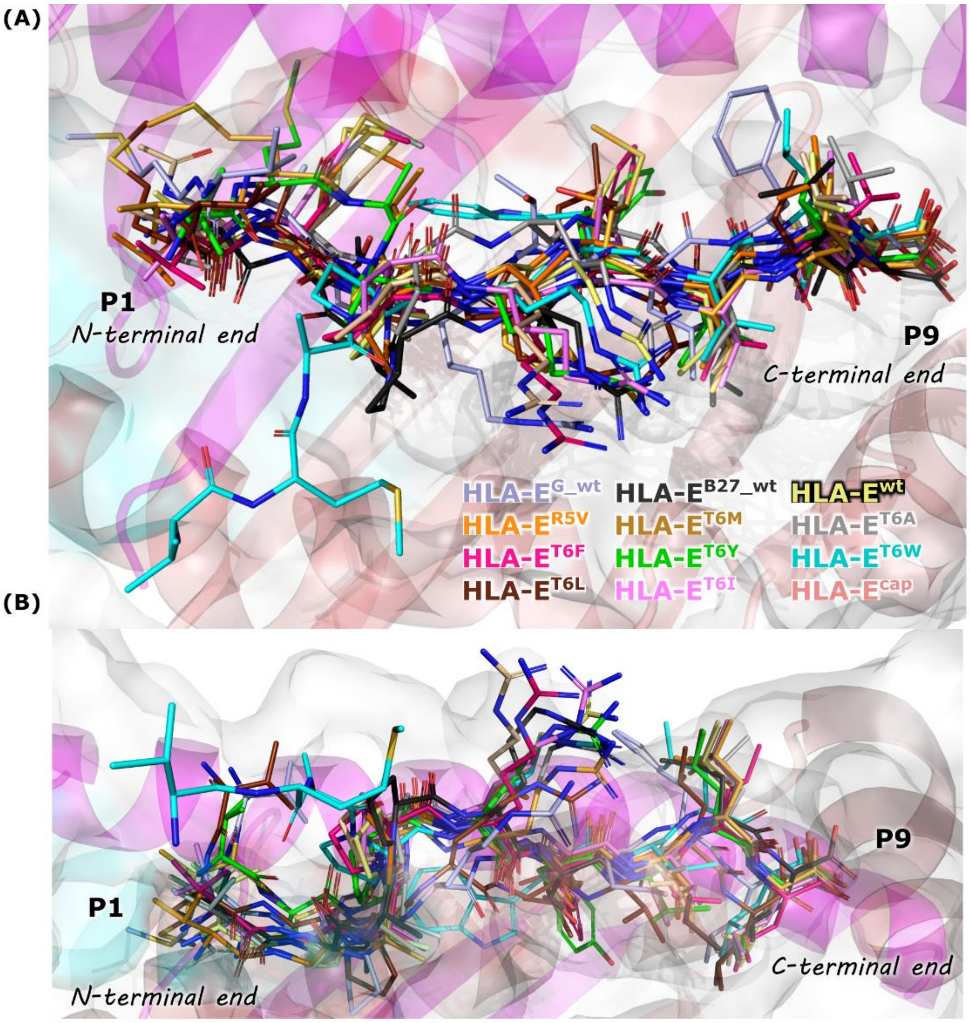

Supplementary Figure 5. Aligned peptides from most representative clusters for models **HLA-E^{G_wt}** **HLA-E^{B27_wt}**, **HLA-E^{wt}**, **HLA-E^{R5V}**, **HLA-E^{T6M}**, **HLA-E^{T6A}**, **HLA-E^{T6F}**, **HLA-E^{T6Y}**, **HLA-E^{T6W}**, **HLA-E^{T6L}**, **HLA-E^{T6I}**, and **HLA-E^{cap}**. Top view (**A**) and side view (**B**). HLA-E α1 (magenta) and α2 (salmon) domains from model **HLA-E^{wt}** are depicted with cartoon representation, while peptides are shown in licorice. The peptide binding groove was selected as HLA-E residues within 10 Å of the peptide in model **HLA-E^{wt}**. Binding site is represented as white surface, with the pockets A in cyan and B in red.

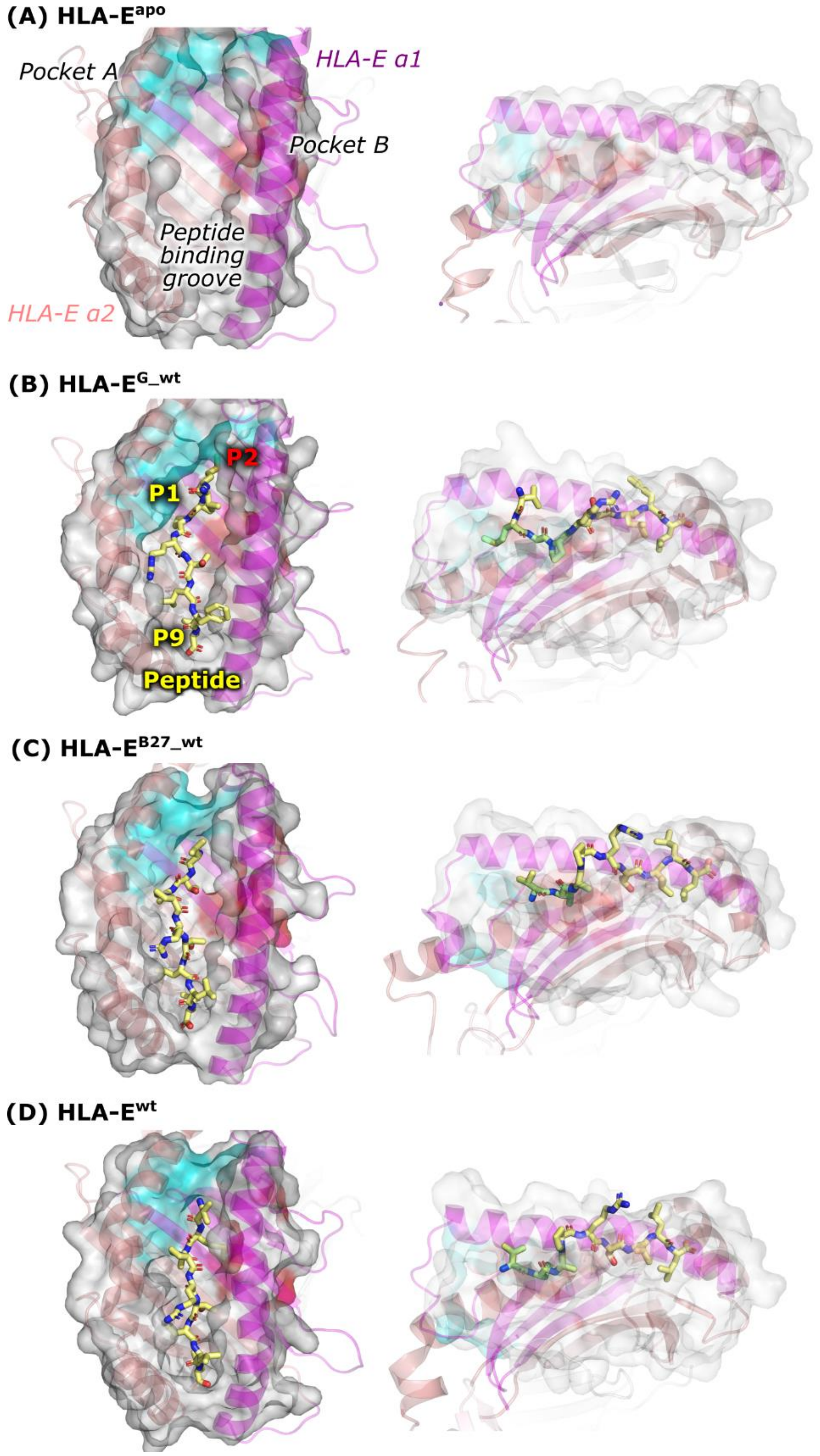

Supplementary Figure 6. The most representative clusters for models (**A**) **HLA-E^{apo}**, (**B**) **HLA-E^{G_wt}**, (**C**) **HLA-E^{B27_wt}**, and (**D**) **HLA-E^{wt}**. Top view on the left panel and side view on the right panel. HLA-E α1 (magenta) and α2 (salmon) domains are depicted with cartoon representation, while peptide (yellow) is shown in licorice. The peptide binding groove was selected as HLA-E residues within 8 Å of the peptide. The binding groove in apo model was determent according to the aligned B7 wild type peptide. Binding site is represented as white surface, with the pockets A in cyan and B in red. In model **HLA-E^{G_wt}** shift of the P2 from pocket B toward pocket A can be seen.

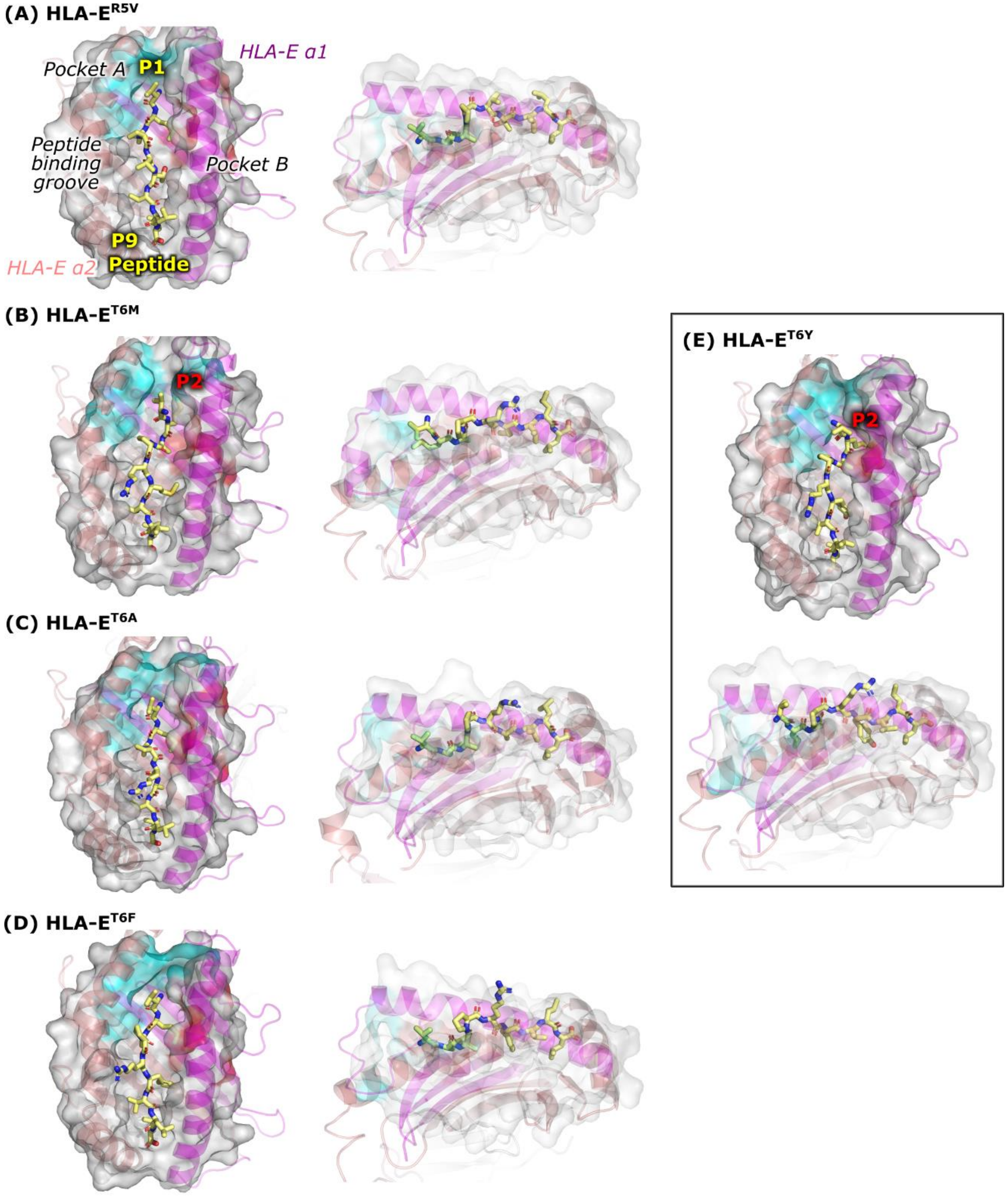

Supplementary Figure 7. The most representative clusters for models (**A**) **HLA-E^{5RV}**, (**B**) **HLA-E^{T6M}**, (**C**) **HLA-E^{T6A}**, (**D**) **HLA-E^{T6F}**, and (**E**) **HLA-E^{T6Y}**. Top view on the left panel and side view on the right panel. HLA-E α1 (magenta) and α2 (salmon) domains are depicted with cartoon representation, while peptide (yellow) is shown in licorice. The peptide binding groove was selected as HLA-E residues within 8 Å of the peptide. Binding site is represented as white surface, with the pockets A in cyan and B in red. In models **HLA-E^{T6M}** and **HLA-E^{T6Y}** shift of the P2 from pocket B toward pocket A can be seen.

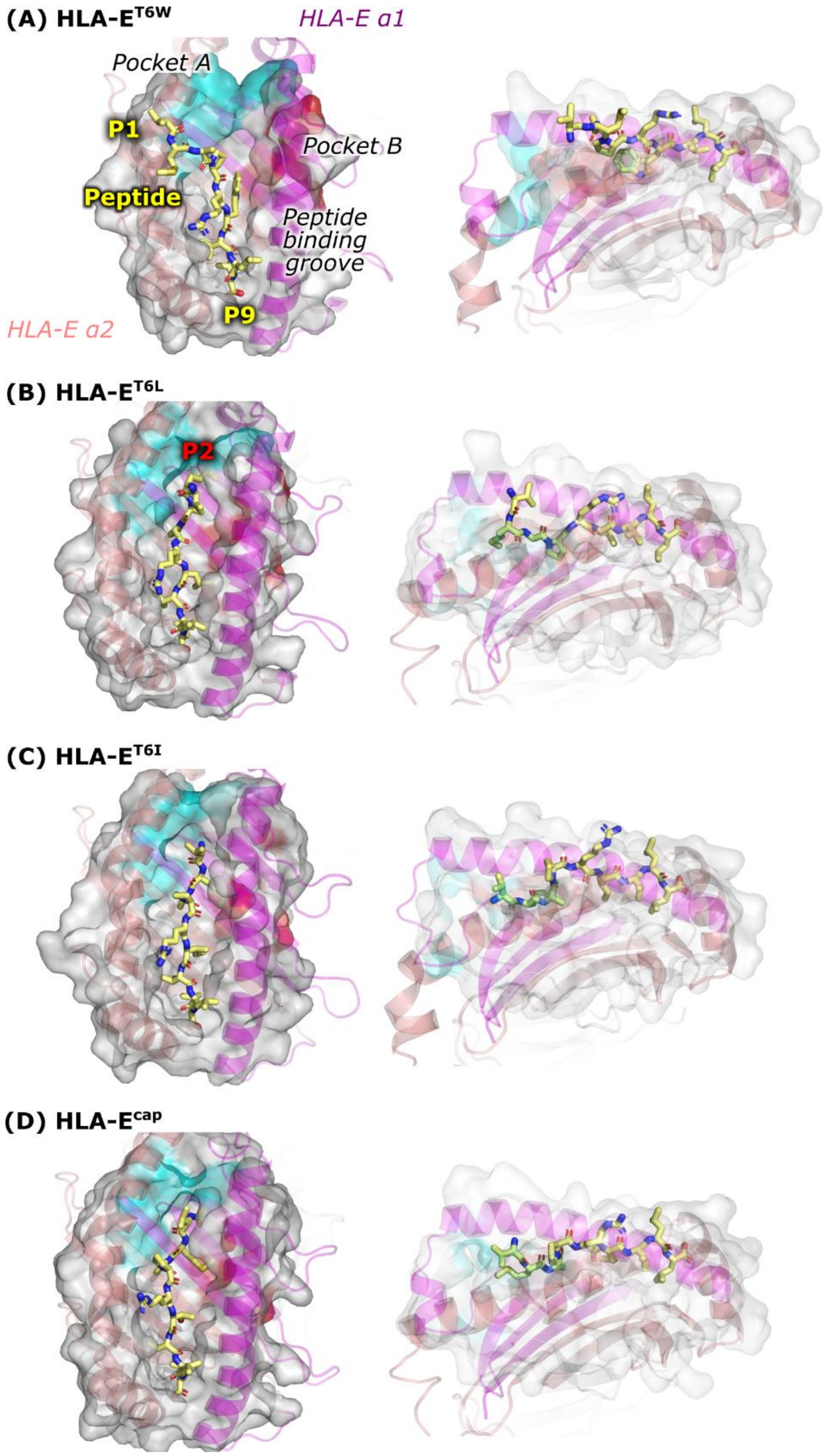

Supplementary Figure 8. The most representative clusters for models (**A**) **HLA-E^{T6W}**, (**B**) **HLA-E^{T6L}**, (**C**) **HLA-E^{T6I}**, and (**D**) **HLA-E^{cap}**. Top view on the left panel and side view on the right panel. HLA-E α1 (magenta) and α2 (salmon) domains are depicted with cartoon representation, while peptide (yellow) is shown in licorice. The peptide binding groove was selected as HLA-E residues within 8 Å of the peptide. Binding site is represented as white surface, with the pockets A in cyan and B in red. In model **HLA-E^{T6L}** shift of the P2 from pocket B toward pocket A can be seen.

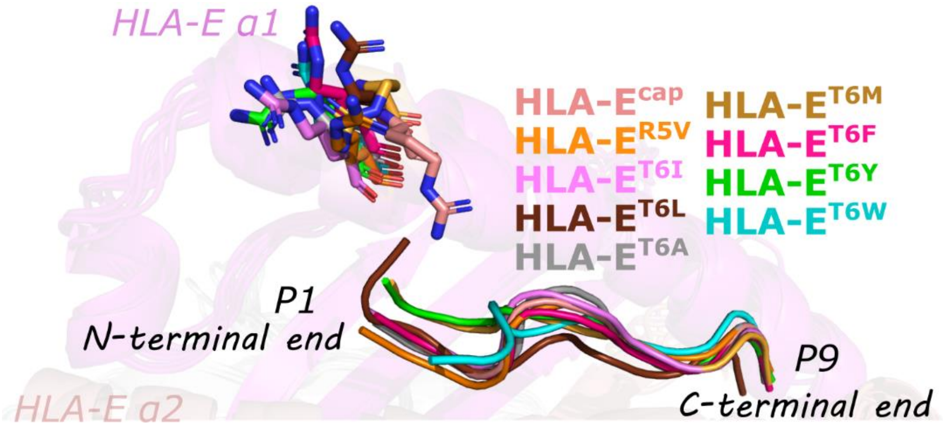

Supplementary Figure 9. Conformations of the Arg62$^{HLA\text{-}E}$ in most representative clusters of **HLA-E^{cap}**, **HLA-E^{R5V}**, **HLA-E^{T6I}**, **HLA-E^{T6L}**, **HLA-E^{T6A}**, **HLA-E^{T6M}**, **HLA-E^{T6F}**, **HLA-E^{T6Y}**, and **HLA-E^{T6W}** models. Compared to other models, Arg62$^{HLA\text{-}E}$ point towards C-terminal in **HLA-E^{cap}** model.

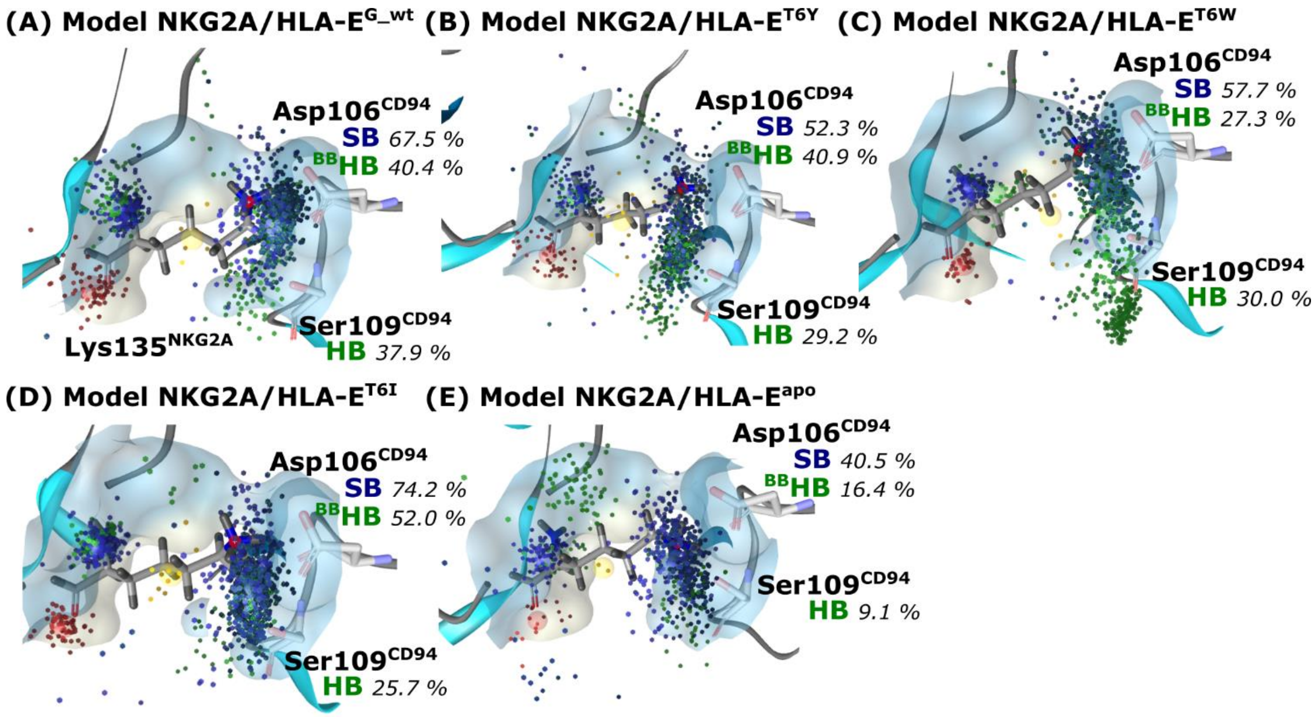

Supplementary Figure 10. Dynophores for Lys135$^{\text{NKG2A}}$ of the immune complexes (**A**) **NKG2A/HLA-E$^{\text{G_wt}}$**, (**B**) **NKG2A/HLA-E$^{\text{T6Y}}$**, (**F**) **NKG2A/HLA-E$^{\text{T6W}}$**, (**G**) **NKG2A/HLA-E$^{\text{T6I}}$** and (**H**) **NKG2A/HLA-E^{apo}** for 1000 uniformly distributed frames between 10 ns and 1 μs of the equilibrated part of the trajectories. The percentage of trajectories in which an interaction is present is given for each model. More condensed interactions correspond to the more stable interaction. SB – salt bridge, HB – hydrogen bond, ^{BB} – the interaction is formed with the backbone atom.

1.2 Supplementary Tables

Supplementary Table 1. Per-residue decomposition of Molecular Mechanics-Generalized Born Surface Area (MM-GBSA) binding free energies (ΔG_b) between peptide and HLA-E/β2m for peptide residues given in kcal/mol.

Peptide	P1	P2	P3	P4	P5	P6	P7	P8	P9
**G_wt**	1.8 ± 1.7	-8.1 ± 1.3	-2.1 ± 0.4	-4.9 ± 0.5	-8.2 ± 1.8	0.2 ± 0.7	-6.0 ± 0.8	-3.1 ± 0.8	-8.2 ± 2.3
**B27_wt**	3.0 ± 2.4	-2.8 ± 1.8	-1.2 ± 1.1	-1.4 ± 0.9	-4.7 ± 2.6	-0.2 ± 0.8	-5.9 ± 0.9	-2.8 ± 0.7	-8.2 ± 2.5
**wt**	0.8 ± 2.3	-9.4 ± 1.3	-3.5 ± 0.7	-1.7 ± 0.4	-6.7 ± 2.0	-1.6 ± 1.6	-5.7 ± 0.8	-3.5 ± 0.7	-7.9 ± 3.1
**R5V**	1.5 ± 2.4	-8.7 ± 1.7	-2.7 ± 1.8	-2.1 ± 0.8	-2.0 ± 1.3	-1.8 ± 1.1	-5.2 ± 0.9	-2.9 ± 0.9	-8.7 ± 2.2
**T6M**	1.5 ± 2.0	-6.9 ± 1.4	-2.0 ± 0.8	-2.3 ± 0.8	-7.6 ± 1.8	-6.5 ± 1.0	-5.5 ± 0.9	-3.2 ± 0.7	-7.0 ± 2.8
**T6A**	2.3 ± 2.5	-7.8 ± 1.3	-2.3 ± 1.3	-2.3 ± 1.2	-7.1 ± 2.3	-1.4 ± 0.6	-5.1 ± 0.8	-2.7 ± 0.7	-8.7 ± 2.2
**T6F**	3.6 ± 2.1	-6.3 ± 1.5	-3.4 ± 1.4	-2.4 ± 0.9	-9.2 ± 2.3	-4.5 ± 1.3	-5.0 ± 0.7	-5.0 ± 1.1	-6.6 ± 1.6
**T6Y**	3.7 ± 2.1	-7.3 ± 1.0	-1.9 ± 0.9	-2.6 ± 0.5	-7.3 ± 1.7	-5.9 ± 1.3	-5.0 ± 0.7	-2.8 ± 0.7	-7.5 ± 2.5
**T6W**	4.0 ± 2.9	-3.9 ± 0.9	-1.1 ± 0.6	-3.4 ± 0.4	-7.0 ± 1.9	-5.4 ± 0.8	-4.9 ± 0.6	-3.3 ± 0.7	-8.5 ± 2.3
**T6L**	3.3 ± 2.3	-9.4 ± 1.4	-3.2 ± 1.0	-5.1 ± 0.7	-9.0 ± 1.5	-5.9 ± 0.7	-6.1 ± 0.6	-3.1 ± 0.7	-8.2 ± 2.1
**T6I**	1.0 ± 2.2	-8.8 ± 0.9	-3.4 ± 0.7	-1.6 ± 0.4	-7.1 ± 3.0	-6.1 ± 0.7	-5.6 ± 0.7	-2.9 ± 0.8	-8.5 ± 2.0
**cap**	-9.8 ± 2.4	-7.3 ± 1.2	-3.3 ± 0.8	-1.1 ± 0.3	-7.8 ± 1.5	-0.7 ± 0.7	-4.5 ± 0.7	-3.1 ± 0.9	-7.9 ± 2.7

Supplementary Table 2. Composition of pockets that accommodate the side chains of peptide residues in HLA-E (O'Callaghan et al., 1998).

Pocket	residue accommodation	HLA-E residues composition
**A**	P1	Thr163, Tyr159, Trp167, Tyr59, Tyr171, Leu168
**B**	P2	Met45, Ala67, Ser24, Phe22, His9
**C**	P6	Ile73, Thr70, Phe74, Trp97, Phe116
**D**	P3	Gln156, Tyr159, Trp97, His99
**E**	P7	Ser147, Trp133, Leu124, Glu114, Phe116
**F**	P9	Leu81, Tyr123, Leu95, Leu124, Phe116

Supplementary Table 3. Per-residue root mean square fluctuations (RMSF) (Å) of peptide variants from the last of 1 µs of the MD simulations of the HLA-E/β2m ligand-only systems. RMSF values are calculated for residues' Cα atoms.

Peptide	P1	P2	P3	P4	P5	P6	P7	P8	P9
**G_wt**	1,35	1,51	1,75	2,23	1,53	0,97	0,91	0,98	1,03
**B27_wt**	2,01	1,89	2,32	2,4	1,65	1,35	1,14	1,19	1,20
**wt**	0,64	0,59	0,64	0,75	0,78	0,78	0,77	0,8	0,79
**R5V**	1,16	1,57	2,12	2,38	1,83	1,2	1,2	1,38	1,51
**T6M**	1,7	1,5	1,45	1,29	1,02	1,02	1,15	1,3	1,34
**T6A**	1,76	1,92	1,83	1,78	1,48	1,22	1,56	1,94	2,12
**T6F**	1,19	1,42	1,44	1,8	1,65	1,32	1,32	1,72	1,55
**T6Y**	1,6	1,23	1,62	1,01	0,98	0,94	1,05	1,03	1,00
**T6W**	3,15	2,18	1,33	1,66	1,06	0,91	1,09	1,24	1,34
**T6L**	1,92	1,53	1,44	1,72	0,99	0,73	0,76	0,87	0,90
**T6I**	0,78	0,66	0,70	0,80	0,79	0,81	0,88	0,98	0,99
**cap**	1,30	0,83	0,84	1,00	0,85	0,76	0,79	0,83	0,85

Supplementary Table 4. a) Binding free energies (ΔG_b) calculated using the molecular mechanics/generalized born surface area (MM-GBSA) method and b) interaction energies (ΔE) calculated using the gmx energy module of the Gromacs2016 between peptides and HLA-E/β2m.

a)

Model	VDW [kcal/mol]	EEL [kcal/mol]	EGB [kcal/mol]	ESURF [kcal/mol]	ΔG_b [kcal/mol]
**HLA-E^{G_wt}**	-83.8 ± 4.3	-472.0 ± 24.7	484.5 ± 20.9	-13.8 ± 0.4	**-85.1 ± 5.9**
**HLA-E^{B27_wt}**	-65.7 ± 6.2	-495.6 ± 35.4	511.3 ± 27.9	-11.2 ± 0.7	**-61.1 ± 9.9**
**HLA-E^{wt}**	-87.5 ± 4.9	-551.7 ± 34.0	553.2 ± 27.4	-14.1 ± 0.4	**-100.0 ± 9.2**
**HLA-E^{R5V}**	-84.0 ± 7.6	-274.0 ± 28.0	287.4 ± 21.5	-12.8 ± 0.9	**-83.5 ± 15.5**
**HLA-E^{T6M}**	-83.1 ± 6.7	-488.5 ± 37.9	498.8 ± 32.8	-13.8 ± 0.7	**-86.6 ± 10.9**
**HLA-E^{T6A}**	-77.2 ± 7.3	-512.1 ± 39.8	518.2 ± 35.8	-13.0 ± 0.8	**-84.0 ± 11.8**
**HLA-E^{T6F}**	-84.5 ± 6.7	-506.6 ± 26.6	521.6 ± 21.2	-13.3 ± 0.7	**-82.9 ± 10.1**
**HLA-E^{T6Y}**	-91.3 ± 5.5	-518.6 ± 32.1	535.9 ± 26.5	-14.5 ± 0.6	**-88.5 ± 10.8**
**HLA-E^{T6W}**	-74.4 ± 5.3	-511.9 ± 39.0	520.7 ± 35.3	-11.9 ± 0.5	**-77.5 ± 6.3**
**HLA-E^{T6L}**	-89.0 ± 5.4	-514.7 ± 26.9	518.1 ± 22.9	-14.5 ± 0.5	**-100.1 ± 6.4**
**HLA-E^{T6I}**	-88.4 ± 5.0	-532.8 ± 33.2	531.8 ± 26.6	-14.0 ± 0.5	**-103.3 ± 8.9**
**HLA-E^{cap}**	-86.2 ± 5.3	-286.6 ± 21.2	298.9 ± 16.9	-13.5 ± 0.6	**-87.5 ± 8.4**

b)

Model	LJ-SR [kJ/mol]	Coul-SR [kJ/mol]	ΔE [kJ/mol]	ΔE [kcal/mol]
**HLA-E^{G_wt}**	-279.9 ± 28.7	-585.2 ± 55.3	-865.1 ± 84.0	**-206.6 ± 20.1**
**HLA-E^{B27_wt}**	-227.7± 23.9	-530.9 ± 67.4	-758.5 ± 91.3	**-181.2 ± 21.8**
**HLA-E^{wt}**	-307.0 ± 22.8	-671.2 ± 68.5	-978.2 ± 91.3	**-233.6 ± 21.8**
**HLA-E^{R5V}**	-295.7 ± 29.3	-491.2 ± 62.3	-786.9 ± 91.6	**-187.9 ± 21.9**
**HLA-E^{T6M}**	-285.1 ± 26.4	-540.4 ± 78.5	-825.5 ± 104.9	**-197.2 ± 25.0**
**HLA-E^{T6A}**	-286.9 ± 32.9	-639.6 ± 78.1	-926.5 ± 110.9	**-221.3 ± 26.5**
**HLA-E^{T6F}**	-302.4 ± 28.1	-585.6 ± 81.5	-887.9 ± 109.6	**-212.1 ± 26.2**
**HLA-E^{T6Y}**	-307.9 ± 30.0	-588.2 ± 81.3	-896.1 ± 111.3	**-214.0 ± 26.6**
**HLA-E^{T6W}**	-250.6 ± 24.1	-545.5 ± 82.2	-796.1 ± 106.4	**-190.1 ± 25.4**
**HLA-E^{T6L}**	-304.2 ± 23.2	-661.6 ± 61.7	-965.8 ± 84.9	**-230.7 ± 20.3**
**HLA-E^{T6I}**	-308.4 ± 22.9	-623.3 ± 60.4	-931.7 ± 83.2	**-222.5 ± 19.9**
**HLA-E^{cap}**	-311.3 ± 24.8	-521.5 ± 67.5	-832.8 ± 92.2	**-198.9 ± 22.0**

2 Supplementary References

O'Callaghan, C. A., Tormo, J., Willcox, B. E., Braud, V. M., Jakobsen, B. K., Stuart, D. I., McMichael, A. J., Bell, J. I., and Jones, E. Y. (1998). Structural features impose tight peptide binding specificity in the nonclassical MHC molecule HLA-E. *Mol. Cell* 1, 531–41. doi:10.1016/s1097-2765(00)80053-2.

Wolber, G., and Langer, T. (2005). LigandScout: 3-D pharmacophores derived from protein-bound ligands and their use as virtual screening filters. *J. Chem. Inf. Model.* 45, 160–169. doi:10.1021/ci049885e.
